# Epigenetic Rejuvenation of Mesenchymal Stromal Cells Derived from Induced Pluripotent Stem Cells

**DOI:** 10.1016/j.stemcr.2014.07.003

**Published:** 2014-08-14

**Authors:** Joana Frobel, Hatim Hemeda, Michael Lenz, Giulio Abagnale, Sylvia Joussen, Bernd Denecke, Tomo Šarić, Martin Zenke, Wolfgang Wagner

**Affiliations:** 1 Helmholtz-Institute for Biomedical Engineering, RWTH Aachen University Medical School, 52074 Aachen, Germany; 2 Aachen Institute for Advanced Study in Computational Engineering Science (AICES), RWTH Aachen University, 52074 Aachen, Germany; 3 Interdisciplinary Center for Clinical Research (IZKF), RWTH Aachen University Medical School, 52074 Aachen, Germany; 4 Center for Physiology and Pathophysiology, Institute for Neurophysiology, University of Cologne, 50931 Cologne, Germany; 5 Institute for Biomedical Engineering, Cell Biology, RWTH Aachen University Medical School, 52074 Aachen, Germany

## Abstract

Standardization of mesenchymal stromal cells (MSCs) remains a major obstacle in regenerative medicine. Starting material and culture expansion affect cell preparations and render comparison between studies difficult. In contrast, induced pluripotent stem cells (iPSCs) assimilate toward a ground state and may therefore give rise to more standardized cell preparations. We reprogrammed MSCs into iPSCs, which were subsequently redifferentiated toward MSCs. These iPS-MSCs revealed similar morphology, immunophenotype, in vitro differentiation potential, and gene expression profiles as primary MSCs. However, iPS-MSCs were impaired in suppressing T cell proliferation. DNA methylation (DNAm) profiles of iPSCs maintained donor-specific characteristics, whereas tissue-specific, senescence-associated, and age-related DNAm patterns were erased during reprogramming. iPS-MSCs reacquired senescence-associated DNAm during culture expansion, but they remained rejuvenated with regard to age-related DNAm. Overall, iPS-MSCs are similar to MSCs, but they reveal incomplete reacquisition of immunomodulatory function and MSC-specific DNAm patterns—particularly of DNAm patterns associated with tissue type and aging.

## Highlights

• MSC-derived iPSCs are redifferentiated toward MSCs in a one-step protocol
• Gene expression profiles of iPS-MSCs closely resemble those of primary MSCs
• DNA methylation (DNAm) profiles of iPS-MSCs lag behind those of MSCs
• Age-related and tissue-specific DNAm patterns remain erased in iPS-MSCs

## Graphical Abstract

**Figure:**
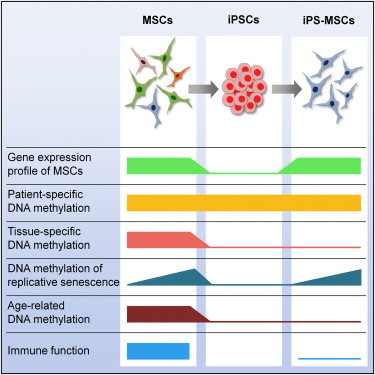

## Teaser

Wagner and colleagues redifferentiated MSC-derived iPSCs toward MSCs. These iPS-MSCs reveal similar morphology, immunophenotype, and in vitro differentiation potential as primary MSCs, but they were impaired in suppressing T cell proliferation. Furthermore, there are marked differences in DNA methylation profiles that can, at least partially, be attributed to persistent reset of tissue-specific and age-related DNA methylation changes.

## Introduction

Mesenchymal stromal cells (MSCs) are heterogeneous cell preparations and only a small subpopulation often referred to as “mesenchymal stem cells” possesses multilineage differentiation potential (Dominici et al., 2006). MSC preparations are greatly affected by starting material, such as bone marrow (BM) or adipose tissue (AT), and cell-culture media. Furthermore, they acquire functional changes during culture expansion ending in replicative senescence (Wagner and Ho, 2007). So far, MSCs are scarcely defined by fibroblastoid plastic adherent growth, a panel of nonspecific surface markers, and their capacity to differentiate toward adipogenic, osteogenic, and chondrogenic lineages (Dominici et al., 2006).

In this regard, induced pluripotent stem cells (iPSCs) converge to a better-defined ground state of pluripotency (Hackett et al., 2013). They can be differentiated into all cell types of the organism and—while in pluripotent state—cultured virtually indefinitely without signs of replicative senescence. Epigenetic profiles, such as DNA methylation (DNAm) patterns, are reorganized during reprogramming of somatic cells into iPSCs and closely resemble those of embryonic stem cells (ESCs) (Huang et al., 2014). In particular, senescence-associated DNAm, which is acquired during in vitro expansion (Koch et al., 2013), and age-related DNAm, which accumulate during aging of the organism (Horvath, 2013), are reversed to ground state. In comparison to primary cells, iPSCs are therefore better defined and offer a good starting point for large-scale generation of standardized derivatives, such as iPSC-derived MSCs (iPS-MSCs).

Several groups described strategies to derive MSC-like cells from either ESCs (Barberi et al., 2005; Boyd et al., 2009) or iPSCs (Liu et al., 2012; Diederichs and Tuan, 2014; Zhang et al., 2011). These approaches were based on coculture with primary MSCs, growth factor combinations, or spontaneous differentiation in embryoid bodies (EBs). So far, it has not been analyzed whether DNAm patterns of iPS-MSCs resemble those of primary MSCs.

## Results

### Redifferentiation of iPSCs toward iPS-MSCs

**Figure 1:**
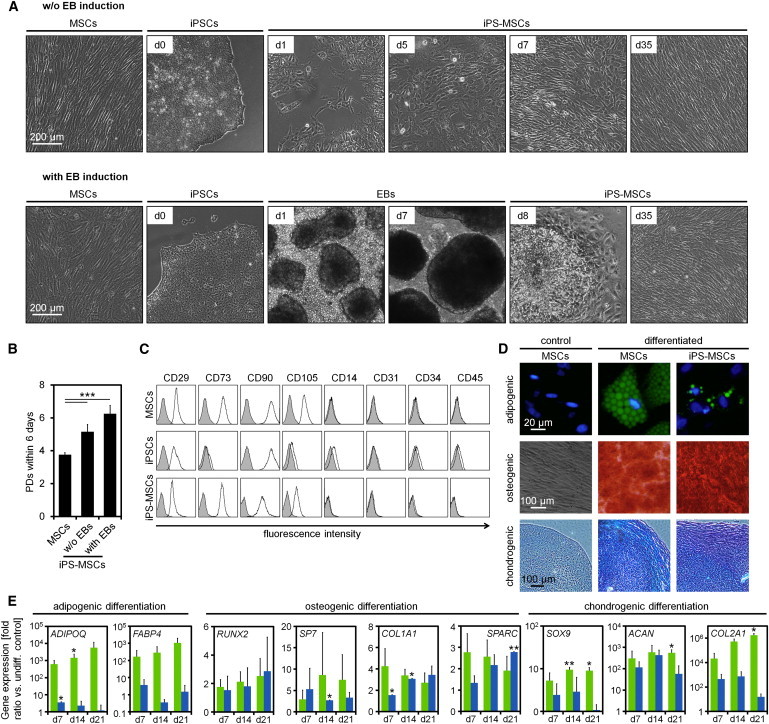
Generation of iPS-MSCs (A) Phase contrast images of MSCs, iPSCs, and iPS-MSCs in the course of differentiation either with or without EB formation. Thirty-five days after induction of differentiation, iPS-MSCs revealed similar fibroblastoid morphology as MSCs. (B) Population doublings (PDs) of MSCs and iPS-MSCs within 6 days of culture on gelatin-coated plates (N = 3; n = 3; mean ± SD; ^∗∗∗^p < 0.001). (C) iPS-MSCs displayed similar immunophenotypic characteristics as primary MSCs (autofluorescence is indicated in gray). (D) MSCs and iPS-MSCs were differentiated toward adipogenic, osteogenic, or chondrogenic lineages for three weeks and subsequently stained with BODIPY/DAPI, alizarin red, or Alcian blue/PAS, respectively. Controls were simultaneously cultured in normal growth medium, and representative images are presented. (E) In vitro differentiation potential was further assessed by quantitative real-time PCR of adipogenic (*ADIPOQ*, *FABP4*), osteogenic (*RUNX2*, *SP7*, *COL1A1*, *SPARC*), and chondrogenic (*SOX9*, *ACAN*, *COL2A1*) marker genes in MSCs (green) and iPS-MSCs (blue; N = 3; n = 2; mean ± SD; ^∗^p < 0.05; ^∗∗^p < 0.01 versus nondifferentiated control). See also Figure S1.

We have recently reprogrammed MSCs from human bone marrow into iPSCs (Shao et al., 2013). These iPSCs were now redifferentiated toward iPS-MSCs using two alternative protocols: (1) the culture medium was simply exchanged to initial MSC-culture medium that comprised 10% human platelet lysate (hPL) or (2) iPSCs were allowed initially to differentiate into EBs in ultralow attachment plates for 7 days in differentiation medium (Figure S1A available online). Thereafter, cells were cultured under standard culture conditions for MSCs with 10% hPL. After 35 days (four passages), the cells revealed a typical fibroblastoid growth pattern; these cells are referred to as iPS-MSCs in this manuscript (Figure 1A). iPS-MSCs passaged on gelatin-coated (Figure 1B) or noncoated (Figure S1B) tissue culture plastic exhibited significantly higher proliferation rates than primary MSCs of the corresponding passage. The immunophenotype of iPS-MSCs was essentially identical to primary MSCs (CD29^+^, CD73^+^, CD90^+^, CD105^+^, CD14^−^, CD31^−^, CD34^−^, and CD45^−^), albeit CD105 was less expressed in iPS-MSCs (Figures 1C and S1C). Furthermore, differentiation of iPS-MSCs toward osteogenic and chondrogenic lineage was equivalent to MSCs. Adipogenic differentiation was also induced in iPS-MSCs, although accumulation of lipid droplets was less pronounced than in primary MSCs (Figures 1D and S1D). These results on in vitro differentiation potential were further validated by upregulation of lineage-specific marker genes (Figure 1E). Taken together, iPS-MSCs fulfilled the minimal criteria for definition of MSCs (Dominici et al., 2006) - even though less prone to adipogenic differentiation. Because both redifferentiation protocols (with or without EB formation) did not reveal significant differences, we used the one-step differentiation protocol without EB formation and with gelatin coating for subsequent experiments.

### iPS-MSCs Reveal Similar Gene Expression as MSCs

**Figure 2:**
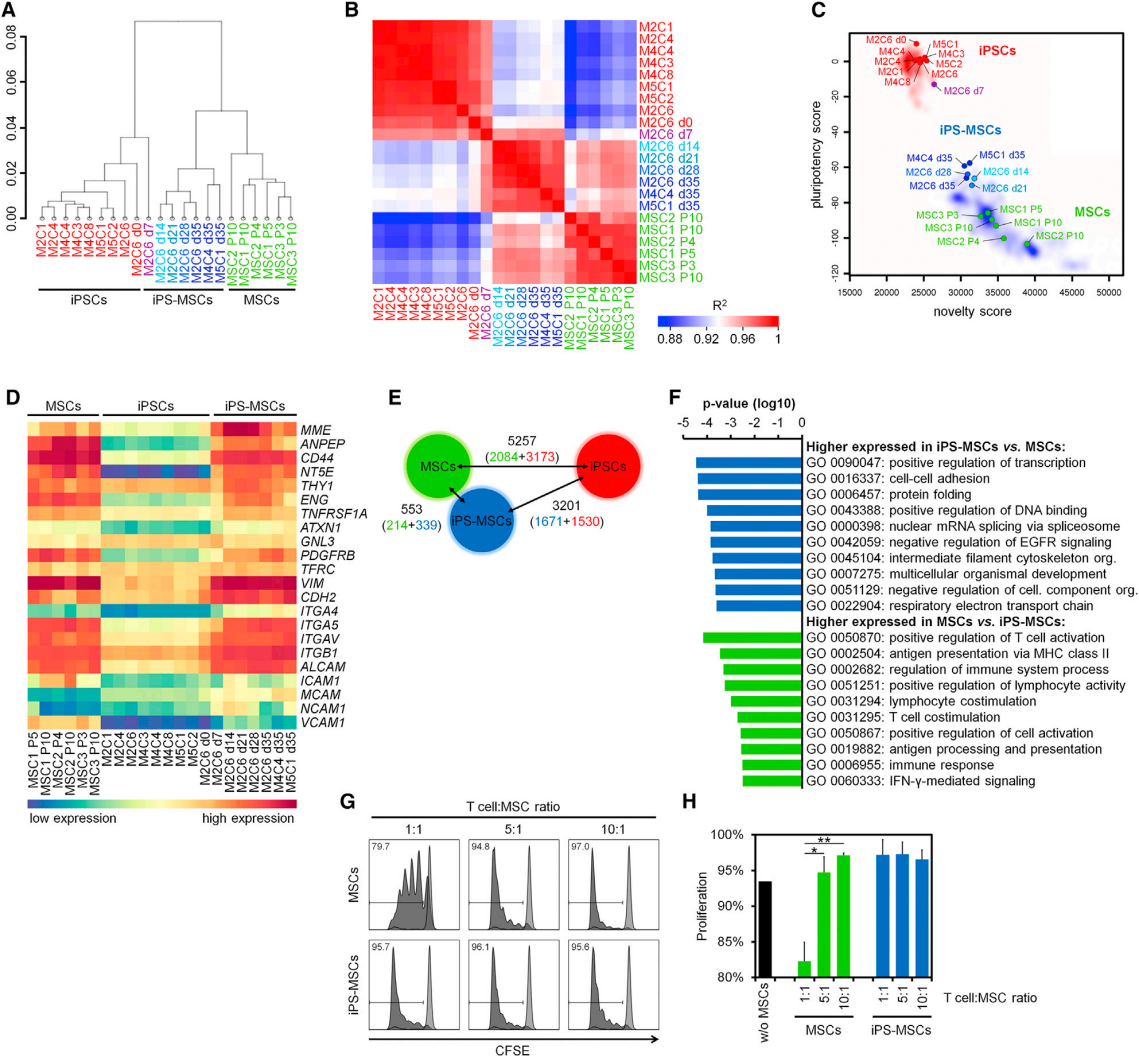
Gene Expression Profiles of iPS-MSCs Are Similar to Primary MSCs (A) Hierarchical clustering revealed close relationship of iPS-MSCs and primary MSCs. MSC donor number (“M”) and clone number (“C”) are indicated for iPSCs and iPS-MSCs. Furthermore, passage numbers (“P”) are provided for MSCs and time of redifferentiation (“d”) for iPS-MSCs. (B) Heatmap of pairwise correlation coefficients (R^2^) demonstrates relationship of iPS-MSCs and MSCs. (C) Pluripotency was assessed by PluriTest analysis (Müller et al., 2011). After differentiation for more than 7 days toward iPS-MSCs, cells were clearly associated with nonpluripotent samples (blue area) and not with pluripotent samples (red area; labeling of samples as in A). (D) MSC marker genes were expressed at similar level in primary MSCs and iPS-MSCs. (E) Number of differentially expressed genes between MSCs, iPSCs, and iPS-MSCs (>2-fold regulation; adjusted p value <0.01; for each cell type, the number of upregulated genes is indicated by color code). (F) Gene ontology analysis of genes that are differentially expressed between MSCs and iPS-MSCs. The most significant categories are depicted. (G) Activity of iPS-MSCs and MSCs on proliferation of stimulated CD4^+^ T cells was assessed by flow cytometry and carboxyfluorescein succinimidyl ester (CFSE) staining. Different T cell:MSC ratios were used and representative histograms are depicted (unstimulated control is indicated in light gray). The percentage of proliferating cells is indicated in each histogram. (H) Quantitative analysis of T cell proliferation assay was performed with percentage of proliferated cells as shown in (G) (MSCs: N = 3; iPS-MSCs: N = 2; mean ± SD; ^∗^p < 0.05; ^∗∗^p < 0.01). See also Figure S2.

Global gene expression was compared in MSCs, iPSCs, and iPS-MSCs. Hierarchical cluster analysis revealed close relationship between iPS-MSCs and MSCs (Figure 2A), which was confirmed by pairwise correlation coefficients (Figure 2B). Gradual changes in gene expression were already observed during the first week of differentiation (Table S1): MSC marker genes including ecto-5′-nucleotidase (*NT5E;* CD73), CD44 antigen (*CD44*), alanyl aminopeptidase (*ANPEP*; CD13), and neural cell adhesion molecule 1 (*NCAM1*; CD56) were already upregulated. On the other hand, pluripotency genes were rapidly downregulated upon differentiation toward iPS-MSCs (Figures 2C, S2A, and S2B). Mesodermal genes typically expressed in MSCs were expressed at a similar level in iPS-MSCs (Figure 2D). Pairwise comparison of gene expression in MSCs, iPSCs, and iPS-MSCs revealed relatively few significantly differentially expressed genes between MSCs and iPS-MSCs (2-fold differential expression and adjusted p value <0.01; Figure 2E; Table S2): 339 genes were higher expressed in iPS-MSCs, and these were particularly enriched in gene ontology (GO) categories of transcriptional regulation, cell adhesion, and development; 214 genes were higher expressed in MSCs that were particularly enriched in GO categories for T cell activation and immune response (Figure 2F). Therefore, we used a surrogate assay to determine suppression of T cell proliferation in coculture with iPS-MSCs or MSCs. Indeed, MSCs significantly suppressed T cell proliferation in a dose-dependent manner, whereas this was not observed in iPS-MSCs, indicating lower immunomodulatory function (Figure 2G). To further classify gene expression profiles of iPS-MSCs, we used PhysioSpace analysis, a bioinformatics tool to interpret gene expression differences between two distinct cell types in terms of physiologically relevant expression patterns (Lenz et al., 2013) that provided further evidence that iPS-MSCs converged toward MSCs (Figure S2C). Overall, gene expression profiles supported the notion that iPS-MSCs closely resemble MSCs, even though there are differences in their immune function.

### DNA Methylation Profiles of iPS-MSCs

**Figure 3:**
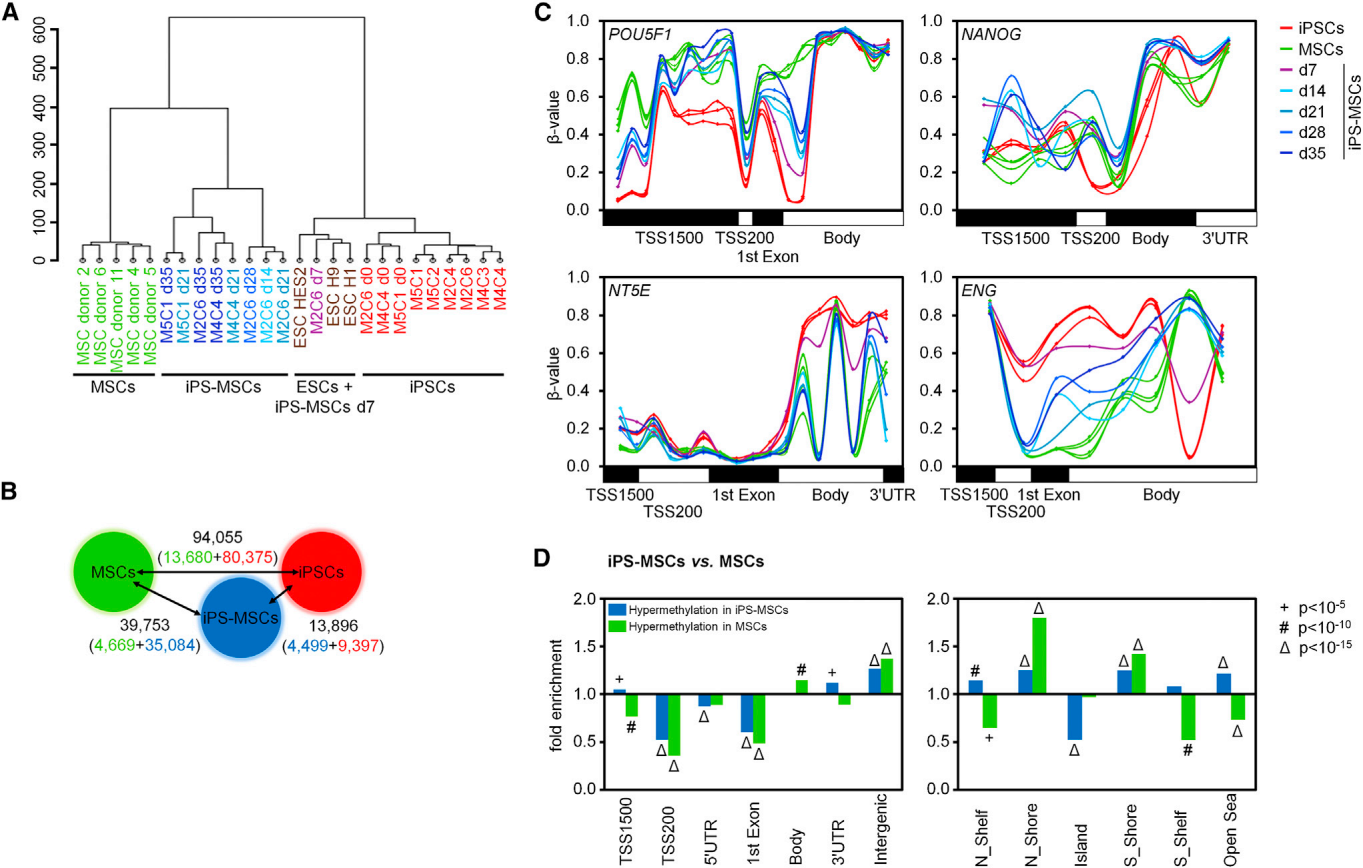
DNAm Profiles of iPS-MSCs (A) Hierarchical clustering of global DNAm profiles. (B) Number of CpGs with differential DNAm between MSCs, iPSCs, and iPS-MSCs (>20% change in DNAm level; adjusted p value <0.01; for each cell type hypermethylated CpGs are indicated by color code). (C) DNAm levels (β values) of CpGs represented in the genes *POU5F1* (OCT3/4), *NANOG*, *NT5E* (CD73), and *ENG* (CD105) (TSS1500: 1,500 bp upstream of transcription start site; TSS200: 200 bp upstream of TSS; UTR). (D) Enrichment of differential DNAm of MSCs versus iPS-MSCs in gene regions or in relation to CpG islands (p values were estimated by hypergeometric distribution). See also Figure S3.

Subsequently, we have analyzed DNAm profiles of MSCs, iPSCs, and iPS-MSCs (each of corresponding donors). Hierarchical clustering demonstrated that iPS-MSCs and MSCs cluster together (Figure 3A). At day 7 of differentiation toward iPS-MSCs the methylome was between pluripotent and nonpluripotent cells (Table S1). However, even after 5 weeks of differentiation 39,753 CpGs remained significantly differentially methylated between iPS-MSCs and MSCs (>20% differential DNAm; adjusted p value <0.01; Table S2), whereas only 13,896 CpGs reached this level of significance in iPS-MSCs versus iPSCs (Figure 3B). Overall, DNAm levels were higher in iPSCs and iPS-MSCs as compared to primary MSCs. Nevertheless, redifferentiation was associated with gradual loss of highly methylated and gain of unmethylated CpG sites (Figure S3A). DNAm was further analyzed in relevant genes – for example hypermethylation of POU class 5 homeobox 1 (*POU5F1*; OCT3/4) and Nanog homeobox gene (*NANOG*), and hypomethylation of surface marker genes *NT5E* (CD73*)* and endoglin (*ENG*; CD105) (Figure 3C). Notably, these DNAm patterns revealed high similarity between primary and redifferentiated MSCs in many genes, particularly in *NT5E*. Comparison of DNAm changes with expression changes of corresponding genes revealed some association, but there was no universal linear correlation (Figures S3B and S3C). Furthermore, DNAm differences of iPS-MSCs and MSCs were enriched in intergenic regions and shore regions of CpG islands (Figures 3D and S3D).

### Comprehensive Analysis of DNAm Changes in iPS-MSCs

**Figure 4:**
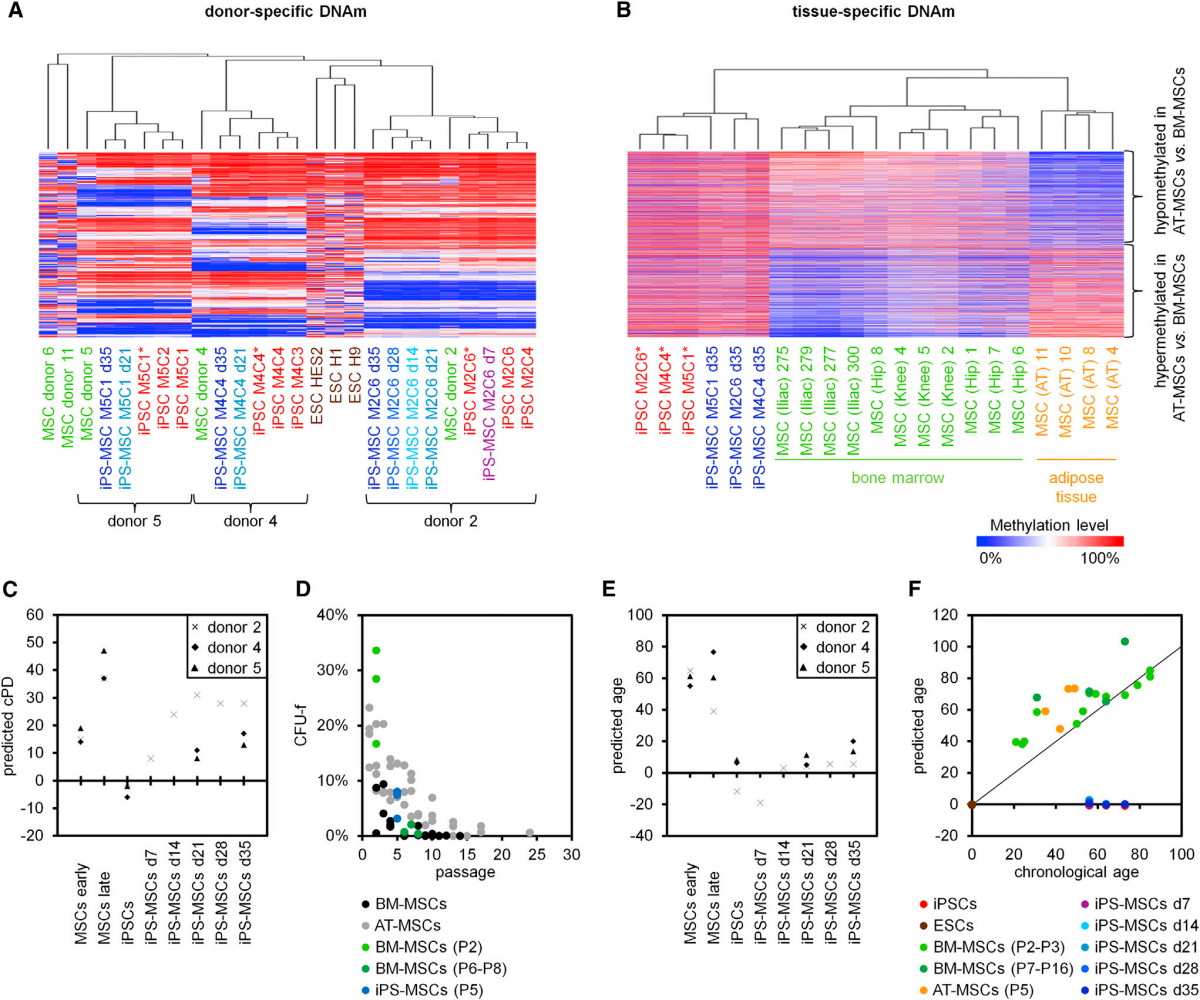
Donor-, Tissue-, and Age-Specific DNAm Changes (A) Hierarchical cluster analysis of 1,091 CpGs with highest donor-specific variation in primary MSC preparations (SD > 0.2) (Shao et al., 2013) revealed that iPSCs and iPS-MSCs clustered with their parental cell preparations. This indicates that interindividual DNAm patterns are maintained in iPS-MSCs (^∗^cultivated in mTeSR1). (B) Hierarchical cluster analysis of 1,711 CpGs with differential DNAm in MSCs from adipose tissue (AT) and bone marrow (BM; >15% difference in mean methylation level) (Schellenberg et al., 2011) demonstrated that the BM-associated DNAm pattern is erased in iPSCs and not reestablished in iPS-MSCs. (C) The state of cellular senescence was estimated by pyrosequencing analysis of six senescence-associated CpGs (Koch et al., 2012). Predictions of this Epigenetic-Senescence-Signature for cumulative population doublings (cPD) were reversed upon reprogramming into iPSCs and increased again during differentiation toward iPS-MSCs. (D) To estimate the state of cellular senescence in iPS-MSCs we analyzed the frequency of fibroblastoid colony forming units (CFU-f). CFU-f frequency declines continuously in primary BM-MSCs and AT-MSCs (Schellenberg et al., 2012) and the number of CFU-f in iPS-MSCs after 35 days is in line with culture expansion for five passages. (E) Donor age of cell preparations was estimated using a multivariate model based on DNAm of 99 age-related CpGs of blood (Weidner et al., 2014). (F) Alternatively, donor age was predicted using a recently published predictor applicable for different tissues (Horvath, 2013). Overall, epigenetic rejuvenation upon reprogramming into iPSCs is also maintained in iPS-MSCs. See also Figure S4.

We have recently demonstrated that iPSCs maintain donor-specific characteristics in their DNAm pattern: 1,091 CpGs with the highest variation in different MSC preparations remained methylated at similar level in corresponding iPSCs (Shao et al., 2013). Here, we demonstrate that this donor-specific pattern was also maintained upon redifferentiation into iPS-MSCs (Figures 4A and S4A).

Subsequently, we analyzed if tissue-specific DNAm patterns are reestablished in iPS-MSCs. We have previously isolated MSCs from adipose tissue (AT) and bone marrow (BM); the latter were either derived from iliac crest (iliac) or caput femoris (hip): 1,711 CpGs revealed at least 15% differential DNAm in AT-MSCs versus BM-MSCs (Schellenberg et al., 2011). These DNAm changes were most significantly enriched in GO categories for nutrient level, lipid modification, and glucose metabolism reflecting functional differences of the originating tissues. Our MSCs from tibia plateau (knee) clustered with the other BM-MSCs. However, this tissue-specific DNAm pattern was erased by reprogramming and not reestablished upon differentiation toward iPS-MSCs (Figure 4B).

Long-term culture of MSCs is associated with highly reproducible DNAm changes—enriched in the homeobox gene cluster (*HOXB*) and the keratin associated protein (KRTAP) locus—which are almost entirely reversed in iPSCs (Koch et al., 2013). Here, we demonstrate that these senescence-associated DNAm changes are regained during culture expansion of iPS-MSCs. In particular CpGs, which are hypomethylated during expansion of MSCs, were reversed to high DNAm levels in iPSCs and subsequently again hypomethylated during expansion of iPS-MSCs (Figure S4B). Alternatively, we estimated cellular senescence by pyrosequencing of six senescence-associated CpGs (Koch et al., 2012). This Epigenetic-Senescence-Signature provides a biomarker that facilitates robust predictions for passage numbers (Figure S4C) and cumulative population doublings (Figures 4C and S4D): senescence predictions increased continuously during differentiation toward iPS-MSCs and after 35 days iPS-MSCs resembled MSCs of early passage in their epigenetic makeup. To gain further insight into the state of cellular senescence of iPS-MSCs, we quantified the frequency of fibroblastoid colony-forming units (CFU-f), which rapidly declines during culture expansion of primary MSCs (Schellenberg et al., 2012). In iPS-MSCs at day 35 (passage 5), about 5% of the cells were capable to form colonies, which correspond to primary MSCs at passage 5 (Figure 4D).

Aging of the organism is also associated with specific DNAm changes, which are reversed upon reprogramming into iPSCs (Horvath, 2013; Weidner et al., 2014). Here, we analyzed whether these age-related DNAm changes are restored in iPS-MSCs. To this end, we have used a set of 99 CpGs, which reveal age-associated DNAm changes in blood. A multivariate model based on these CpGs has been used to estimate donor age (Weidner et al., 2014). In fact, iPS-MSCs reflect moderate accumulation of age-related DNAm but the samples were estimated much younger than the MSC donors (Figure 4E). Similar results were observed using the epigenetic predictor described by Horvath, which is based on more CpGs and which is applicable to different tissues and cell types (Horvath, 2013) (Figure 4F; correlation between the two predictors: R = 0.80). Overall, iPS-MSCs remained rejuvenated with regard to their DNAm profiles.

## Discussion

Induced pluripotent stem cells are a very good basis for generation of standardized cell types. However, differentiation to specific cell types remains a major challenge. Here, we describe a simple protocol for differentiation of iPSCs toward iPS-MSCs, which may be assisted by the fact that our iPSCs were initially derived from MSCs. Furthermore, the medium used for iPS-MSC induction is the same as for initial culture isolation of MSCs. These culture conditions would be compatible with guidelines of good manufacturing practice (GMP) in cellular therapy. Our iPS-MSCs fulfilled the minimal criteria of MSCs (Dominici et al., 2006), but the propensity for adipogenic differentiation was markedly decreased as compared to primary MSCs. This has also been observed by several other groups that used different protocols for generation of iPS-MSCs (Boyd et al., 2009; Chen et al., 2012; Diederichs and Tuan, 2014). Furthermore, adipogenic differentiation is also quite heterogeneous within primary MSCs (Schellenberg et al., 2012). Hence, a more sophisticated molecular definition is required for MSCs and for iPS-MSCs.

MSCs and iPS-MSCs revealed close relationship in gene expression profiles. However, genes associated with T cell activation and immune response were higher expressed in MSCs. Consistent with these observations, iPS-MSCs were impaired in suppressing T cell proliferation, indicating that iPS-MSCs have lower immunomodulatory properties than primary MSCs. Other authors indicated that ESC- and iPSC-derived MSCs are somewhat immunoprivileged and might even have therapeutic efficacy in autoimmune disorder models (de Peppo et al., 2010; Fu et al., 2012; Kimbrel et al., 2014). Therefore, the immunomodulatory function of iPS-MSCs—which is critical for clinical application—deserves further analysis in future studies.

Gene expression changes are not necessarily reflected on DNAm level and vice versa. In fact, many recent studies demonstrated no general correlation of DNAm and gene expression (Wagner et al., 2014), despite the common perception of hypermethylation in promoter regions should entail downregulation of gene expression. Although our understanding of the functional relevance of specific DNAm changes is so far limited epigenetic profiles are very well suited to classify cell preparations: DNAm can be provided as absolute β values at single base resolution; it is relatively stable; less prone to growth conditions; and less influenced by subpopulations, which may highly overexpress subsets of genes. In this regard, the large number of differentially methylated CpGs indicates that iPS-MSCs are rather “MSC-like” than direct correlates of primary MSCs—this has to be taken into account, but it does not exclude that iPS-MSCs may be valuable tools for cellular therapy, too.

We have demonstrated that donor-specific DNAm patterns are maintained upon reprogramming into iPSCs (Shao et al., 2013), and these also remain upon redifferentiation into iPS-MSCs. Thus, there is some epigenetic memory after chromatin remodeling—whether these donor-specific DNAm patterns are functionally relevant remains to be elucidated. On the other hand, tissue-specific epigenetic differences were erased during reprogramming and not reestablished in iPS-MSCs. This may explain some of the epigenetic discrepancy of MSCs and iPS-MSCs.

DNAm changes that accumulate during in vitro culture of MSCs (Koch et al., 2013) are also induced at a similar level during culture expansion of iPS-MSCs—apparently starting with loss of the pluripotent state. In contrast, age-related DNAm, which accumulates during aging of the organism (Weidner et al., 2014; Horvath, 2013) remains overall reset in iPS-MSCs. Notably, epigenetic rejuvenation does not counteract mutations, which may accumulate during in vitro culture. So far, the functional relevance of age-related DNAm changes and the underlying mechanism are not known, but the finding that they remain reset in iPS-MSCs is interesting and encourages further comparison with MSCs from different aged donors in vitro and in vivo. If age-related modifications contribute to loss of regenerative potential this may suggest higher regenerative potential of iPS-MSCs, which may also be reflected by the higher proliferation rates of iPS-MSCs as compared to primary MSCs.

Taken together, we described a simple one-step protocol to redifferentiate MSC-derived iPSCs toward MSCs. These iPS-MSCs reveal similar morphology, immunophenotype, and in vitro differentiation potential as primary MSCs; yet, there are marked differences in DNAm profiles that can, at least partially, be attributed to persistent reset of tissue-specific and age-related DNAm changes. In this regard, iPS-MSCs seem to provide more standardized cell products than primary MSCs, but the therapeutic efficiency—particularly with regard to their immunomodulatory functions—needs to be critically assessed.

## Experimental Procedures

A detailed description of all materials and methods is presented in the Supplemental Experimental Procedures.

### Generation of iPS-MSCs

MSCs were isolated from bone marrow and reprogrammed in to iPSCs as described previously (Shao et al., 2013) (Table S3). For redifferentiation of iPSCs toward iPS-MSCs, we used two alternative strategies: (1) medium was simply exchanged for MSC standard medium with 10% hPL for 7 days, and cells were then further passaged in culture wells with 0.1% gelatin (Sigma-Aldrich) or noncoated plates, or (2) EBs were generated for 7 days in ultralow attachment plates (Corning) and then cultured on either gelatin-coated or noncoated plates.

### Gene Expression Analysis

Gene expression profiles were analyzed by GeneChip Human Gene 1.0 ST Array (Affymetrix).

### DNA Methylation Analysis

DNAm profiles were analyzed using the Infinium HumanMethylation450 BeadChip (Illumina), which addresses 485,577 CpG dinucleotides at a single-nucleotide resolution.

### Statistical Analysis

Results are provided as mean ± SD of at least three independent experiments if not otherwise stated, and Student’s t test was adopted to estimate statistical significance. “N” indicates the number of independent experiments, whereas “n” provides the number of technical replicates within the same experiment.

## Accession Numbers

Gene expression and DNAm data have been deposited at NCBI’s Gene Expression Omnibus (http://www.ncbi.nlm.nih.gov/geo/) under accession numbers GSE54766, GSE38806, GSE46019 (gene expression) and GSE54767, GSE34688, GSE26519, GSE17448 (DNAm).

## Supplemental Information

Document S1. Supplemental Experimental Procedures and Figures S1–S4

Table S1. Differential Gene Expression and DNA Methylation in iPSCs versus iPS-MSCs Day 7

Table S2. Differential Gene Expression and DNA Methylation in iPS-MSCs versus MSCs

Table S3. Information on MSCs Used in This Study

Document S2. Article plus Supplemental Information
