# Supplementary material for: Epigenetic Rejuvenation of Mesenchymal Stromal Cells Derived from Induced Pluripotent Stem Cells
Source: Stem Cell Reports. 2014 Aug 14;3(3):414–22. doi: 10.1016/j.stemcr.2014.07.003 (PMC4266008; doi:10.1016/j.stemcr.2014.07.003)
Supplement: Document S1. Supplemental Experimental Procedures and Figures S1–S4 [file mmc1.pdf]

Stem Cell Reports, Volume *3*

Supplemental Information

Epigenetic Rejuvenation of Mesenchymal Stromal Cells Derived from Induced Pluripotent Stem Cells

Joana Frobel, Hatim Hemeda, Michael Lenz, Giulio Abagnale, Sylvia Joussen, Bernd Denecke, Tomo Šarić, Martin Zenke, and Wolfgang Wagner

Table of Content

Figure S1. Generation and characterization of iPS-MSCs, related to Figure 1. **2**
Figure S2. Down-regulation of pluripotency genes in iPS-MSCs, related to Figure 2. **3**
Figure S3. Global DNAm changes upon re-differentiation, related to Figure 3. **4**
Figure S4. Donor-specific and senescence-associated DNAm in iPS-MSCs, related to Figure 4. **5**
Supplemental Experimental Procedures **6**
Culture of mesenchymal stromal cells 6
MSC-derived induced pluripotent stem cells 6
Generation of iPS-MSCs in Supplemental Information 6
Proliferation analysis (Figure 1B, S1B and S4D) 6
Immunophenotypic analysis (Figure 1C and S1C) 6
In vitro differentiation toward mesodermal lineages (Figure 1D and S1D) 7
Quantitative RT-PCR for mesodermal lineage marker genes (Figure 1E) 7
Immunofluorescent staining (Figure S2A) 7
T cell proliferation assay (Figure 2G and 2H) 7
Gene expression analysis in Supplemental Information (Figure 2A-F, S2B-C and S3B-C) 7
DNAm analysis in Supplemental Information (Figure 3, 4A-C, 4E-F, S3 and S4A-C) 8
Fibroblastoid colony-forming unit (CFU-f) assay (Figure 4D) 8
Supplemental References **8**

Table S1. Differential gene expression and DNA methylation in iPSCs vs. iPS-MSCs day 7 (separate file)
Table S2. Differential gene expression and DNA methylation in iPS-MSCs vs. MSCs (separate file)
Table S3. Information on MSCs used in this study (separate file)

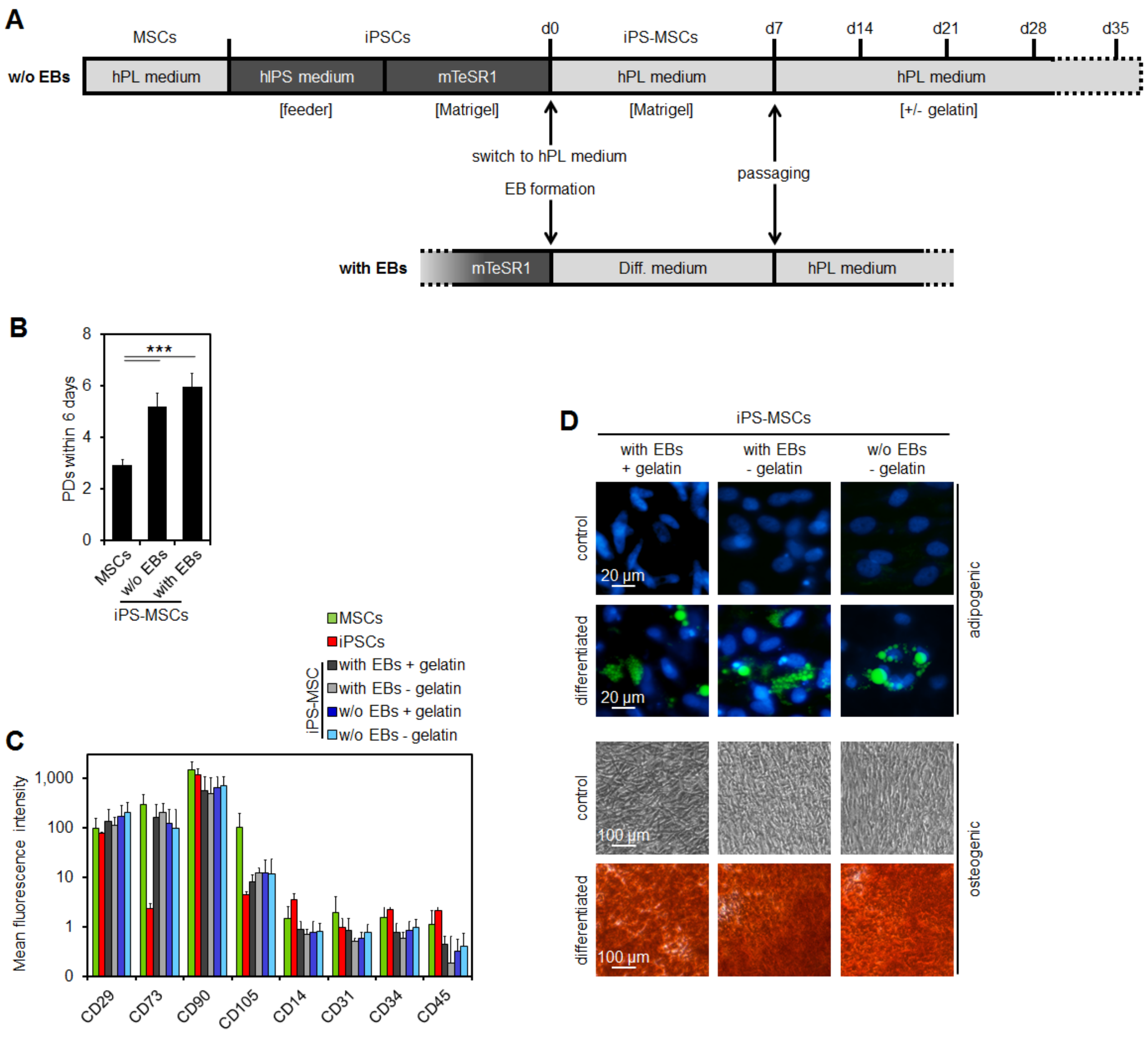

Figure S1. Generation and characterization of iPS-MSCs, related to Figure 1.

(A) Schematic representation of differentiation protocols towards iPS-MSCs. Human bone marrow-derived MSCs were reprogrammed into iPSCs, adopted to feeder-free growth conditions, and further differentiated towards iPS-MSCs using protocols either with or without embryoid body (EB) formation. Differentiation was induced with human platelet lysate (hPL) containing medium and iPS-MSCs were cultivated for five weeks with cell passages once per week.

(B) Population doublings (PDs) of iPS-MSCs and MSCs (both passage 4) on non-coated tissue culture plastic within 6 days (N = 3; n = 3; ± SD; *** $p < 0.001$).

(C) Flowcytometric comparison of MSCs, iPSCs, and iPS-MSCs. Quantitative analysis of data shown in Figure 1C is presented as mean fluorescence intensity normalized to autofluorescence (N ≥ 3; ± SD).

(D) Adipogenic and osteogenic differentiation of MSCs and iPS-MSCs was compared after three weeks of differentiation: osteogenic differentiation (lower panel) was very similar, whereas fat droplet formation in adipogenic differentiation of iPS-MSCs (upper panel) was less pronounced, irrespective of differentiation protocol.

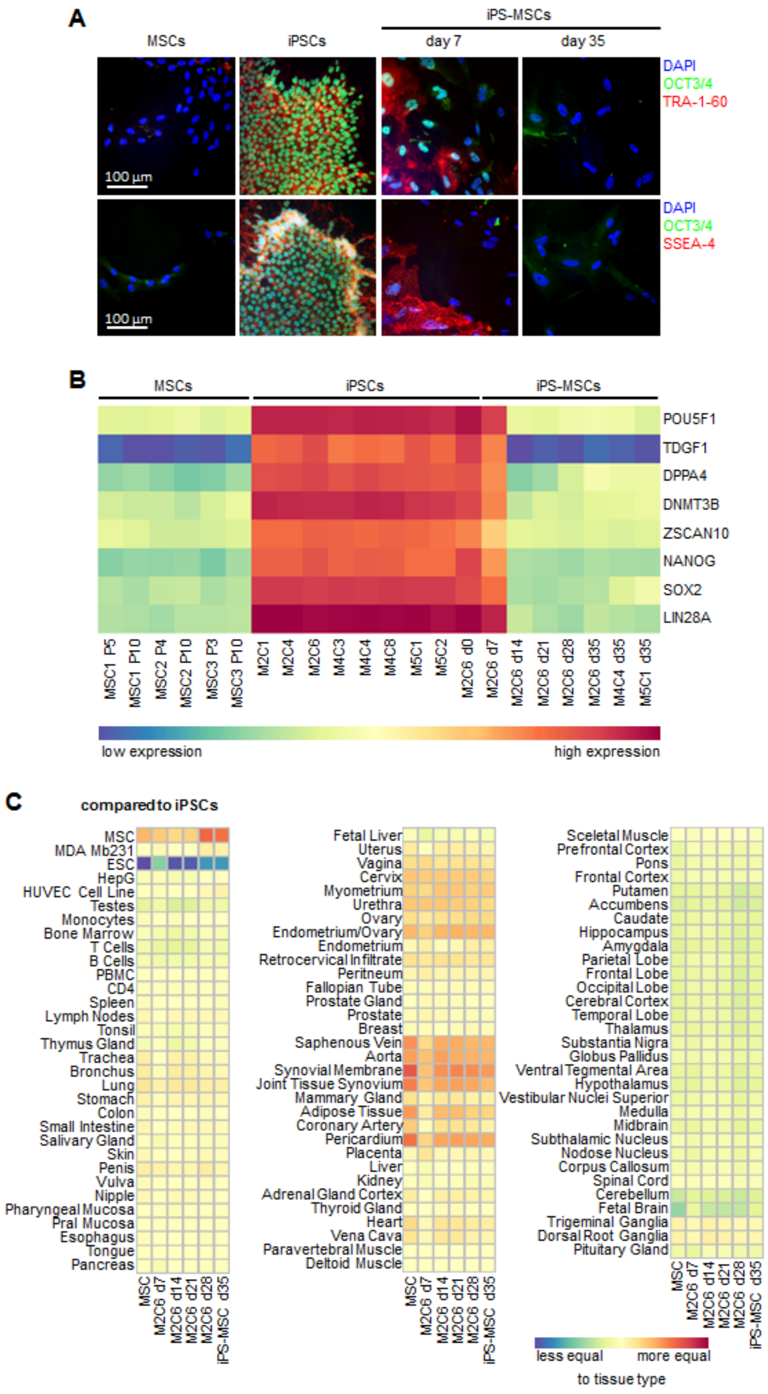

Figure S2. Down-regulation of pluripotency genes in iPS-MSCs, related to Figure 2.

(A) Pluripotency markers OCT3/4, TRA-1-60, and SSEA-4 were stained in MSCs, iPSCs, and iPS-MSCs. Seven days after differentiation towards iPS-MSCs there was still a mixed population of pluripotent and non-pluripotent cells. Size bars 100 µm.

(B) Heatmap of pluripotency gene expression (Affymetrix GeneChip analysis; M = MSC donor number; C = clone number; P = passage number; d = days of differentiation towards iPS-MSCs). Expression of pluripotency genes was down-regulated after 14 days of differentiation. *POU5F1* (OCT3/4); *TDGF1*: Teratocarcinoma-derived growth factor 1; *DPPA4*: Developmental pluripotency associated 4; *DNMT3B*: DNA (cytosine-5-) methyltransferase 3 beta; *ZSCAN10*: Zink finger protein 206; *NANOG*: Nanog; *SOX2*: Sex determining region Y box 2; *LIN28A*: Lin-28 homolog A.

(C) PhysioSpace analysis of MSCs and iPS-MSCs at different days of re-differentiation in comparison to iPSCs showed similarities (red) or differences (blue) to various tissue types. PhysioSpace is a bioinformatics method based on genome-wide tissue-specific gene expression patterns: differential gene expression of two distinct cell types is compared with tissue-specific expression patterns (Lenz et al., 2013). Notably, iPS-MSCs gradually became more MSC-like from day 7 to day 35 of re-differentiation, whereas similarities to ESC-patterns got lost. Furthermore, there was a prominent association of MSCs and iPS-MSCs to tissue-types containing high contents of connective-tissue, such as “Saphenous Vein”, “Aorta”, “Synovial Membrane”, “Joint Tissue Synovium”, or “Pericardium”, as well as to “Adipose Tissue” which contains MSCs. Overall, MSCs and iPS-MSCs showed the same tissue-affiliations.

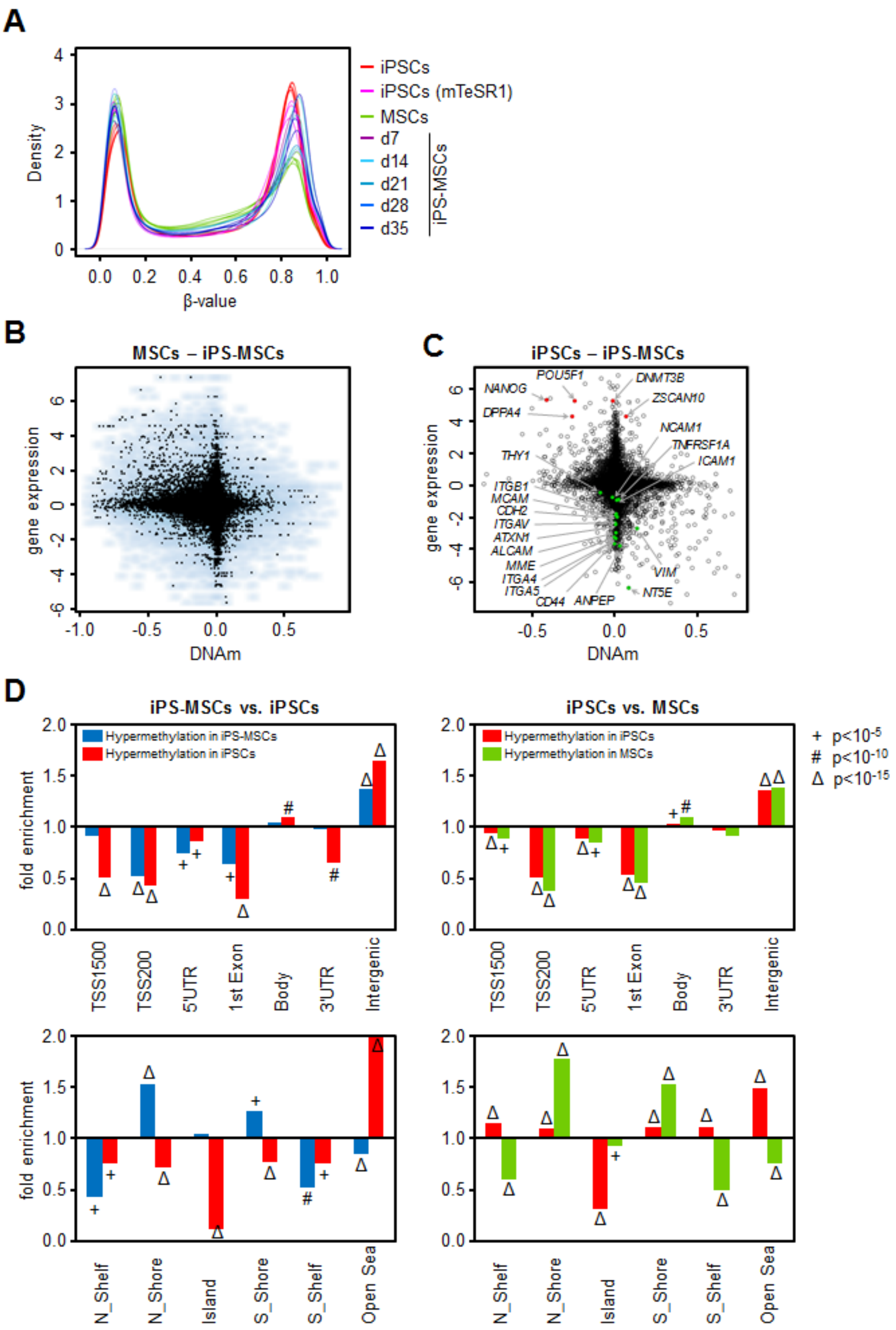

Figure S3. Global DNAm changes upon re-differentiation, related to Figure 3.

(A) Distribution of β-values over all CpG sites in MSCs, iPSCs, and iPS-MSCs. iPSCs were either cultivated on feeder cells (red) or in feeder-free conditions (mTeSR™1, pink).

(B) DNAm changes were analyzed in relation to gene expression changes of corresponding genes (MSCs *versus* iPS-MSCs). There was no clear negative correlation when considering all CpG sites (blue; Pearson correlation: -0.07) and only moderate association for CpG sites located within 200 base pairs up-stream of the transcription start site (TSS200; black dots; Pearson correlation: -0.16).

(C) Comparison of DNAm and gene expression of iPSCs *versus* iPS-MSCs demonstrated high negative correlation for several pluripotency associated genes (red: *NANOG*, *POU5F1*, *DPPA4*), whereas no negative correlation was observed for MSC marker genes (green). β-values of CpGs located within the TSS200 were averaged to be associated with each specific gene only once.

(D) Enrichment of DNAm changes in specific gene regions or in relation to CpG islands (either iPS-MSCs *versus* iPSCs, or iPSCs *versus* MSCs; p-values were estimated by hypergeometric distribution).

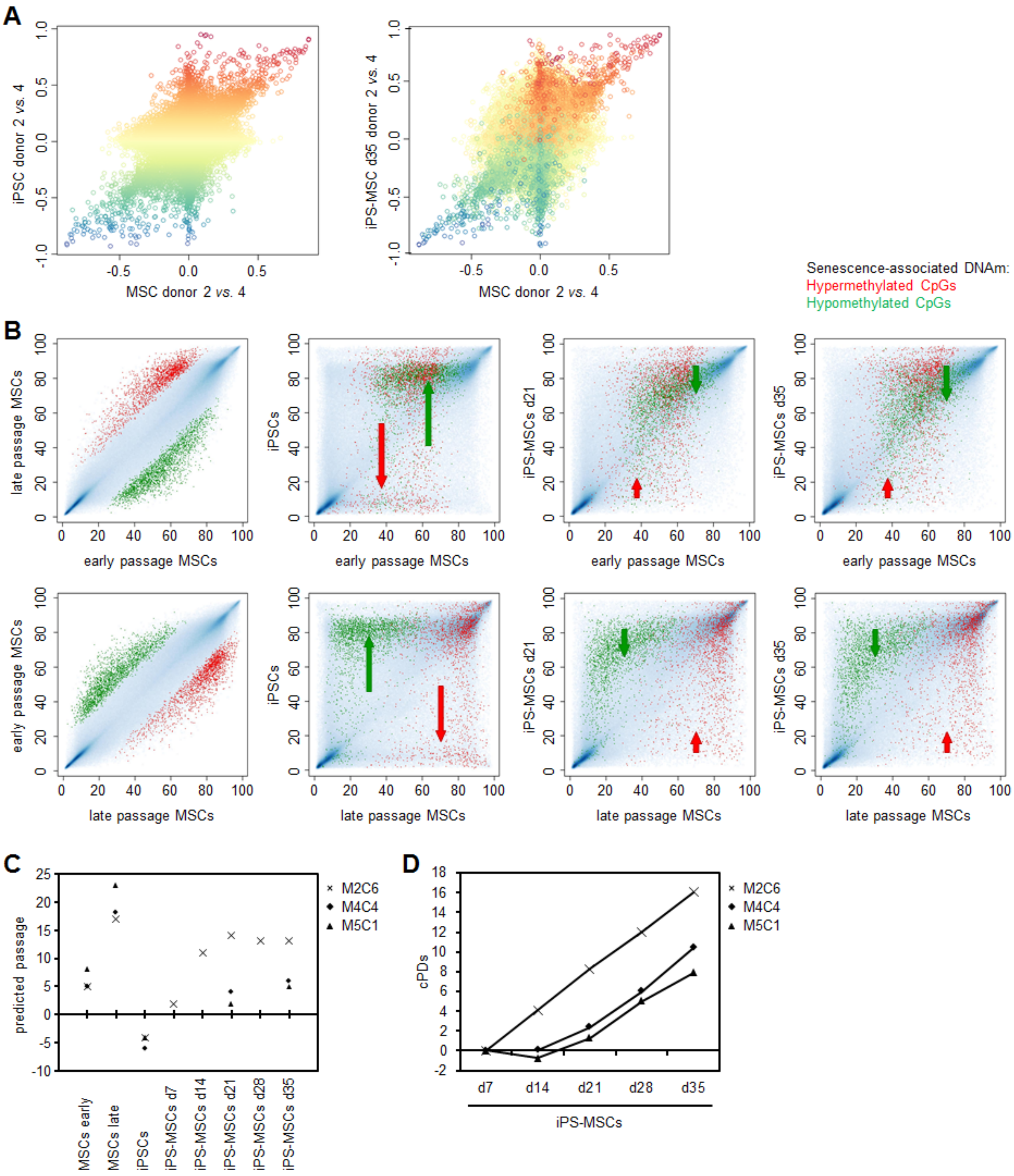

Figure S4. Donor-specific and senescence-associated DNAm in iPS-MSCs, related to Figure 4.

(A) Inter-individual comparison of DNAm profiles in MSCs *versus* iPSCs and MSCs *versus* iPS-MSCs (comparison of donors 2 and 4 is exemplarily depicted). Patient-specific DNAm patterns (left and right in the diagrams) were maintained upon reprogramming into iPSCs and also after re-differentiation towards iPS-MSCs. Furthermore, DNAm differences upon reprogramming (indicated by color code) were also maintained upon re-differentiation into iPS-MSCs.

(B) DNAm (numbers given in % methylation) of MSCs of early (P2-P5) *versus* late passage (P10) reveals CpGs which are hyper- or hypomethylated during culture expansion (depicted in red or green, respectively). DNAm at these senescence-associated CpGs is then presented in iPSCs or iPS-MSCs (either in comparison to MSCs of early passage (upper panel) or MSCs of late passage (lower panel)). Shifts in DNAm are indicated with red and green arrows, respectively.

(C) Cellular senescence was estimated by DNAm at six specific CpG sites using pyrosequencing (Koch et al., 2012). The results indicate that cellular senescence is reset in iPSCs and increased again during culture-expansion of iPS-MSCs.

(D) Cumulative population doublings (cPDs) of iPS-MSCs from three different donors (without EB-formation, with gelatin coating) were assessed upon induction of differentiation for 35 days. Overall, cPDs correlate with predictions for cPDs based on the Epigenetic-Senescence-Signature (Figure 4C).

Supplemental Experimental Procedures

Culture of mesenchymal stromal cells

MSCs were isolated from bone marrow (tibia plateau) of patients undergoing orthopedic surgery after written consent using guidelines approved by the Ethic Committee of the Use of Human Subjects at the University of Aachen (permit number: EK128/09). MSCs were culture-expanded in standard medium consisting of Dulbecco's Modified Eagle Medium (DMEM; 1 g/l glucose; PAA, Pasching, Austria), 1% L-glutamine (PAA), 1% penicillin/streptomycin (PAA), and 10% pooled human platelet lysate (hPL) as previously described (Horn et al., 2010). 0.1% heparin (5,000 IU/ml; Ratiopharm, Ulm, Germany) was added as anticoagulant (Hemeda et al., 2013). Upon 80% confluent growth, cells were reseeded at a density of 5,000 cells/cm^2. For comparison, we used data of adipose-tissue (AT) derived MSCs as described in detail before (Schellenberg et al., 2011; Schellenberg et al., 2012).

MSC-derived induced pluripotent stem cells

iPSCs were generated by infection of MSCs with retroviruses encoding the human transcription factors OCT3/4, SOX2, KLF4, and c-MYC as described previously (Takahashi et al., 2007). Briefly, MSCs were obtained from three different donors (2, 4, and 5) and infected at third passage with pMXs-based retroviruses (Addgene, Cambridge, MS, USA; (Shao et al., 2013). To exclude contamination of feeder cells, iPSCs were adjusted to a feeder-free system on matrigel (BD Biosciences, San Jose, CA, USA) in mTeSR™1 medium (Stemcell Technologies, Vancouver, BC, Canada) for at least three passages.

Generation of iPS-MSCs in Supplemental Information

Derivation of MSCs from iPSCs has been described by many groups using other procedures for differentiation (Chen et al., 2012; Giuliani et al., 2011; Hynes et al., 2013; Lian et al., 2010; Liu et al., 2013; Miao et al., 2014; Moslem et al., 2013; Sun et al., 2012; Tang et al., 2014; TheinHan et al., 2013). In this study, two differentiation strategies for re-differentiation of iPSCs towards iPS-MSCs were used:
Without EBs: Induction of iPS-MSCs was performed by a simple medium switch from mTeSR™1 medium to standard hPL medium. Cells were cultivated for 7 days on Matrigel, and then passaged into gelatin-coated or non-coated plates.
With EBs: For generation of EBs, iPSCs were detached with collagenase and transferred into ultra-low attachment plates. EBs were cultured for 7 days in differentiation medium consisting of Knockout-DMEM (Gibco, Carlsbad, CA, USA) supplemented with 20% FCS (Lonza, Basel, Switzerland), 1% L-glutamine, 1% penicillin/streptomycin, and 0.1 mM β-mercaptoethanol (all Gibco). Subsequently, EBs were seeded into culture plates either coated with 0.1% gelatin or without additional coating and cultured in standard hPL medium.
In the course of differentiation towards iPS-MSC the cells were passaged every 7 days with Trypsin-EDTA (Gibco), and reseeded in a density of 5,000 cells/cm^2.

Proliferation analysis (Figure 1B, S1B and S4D)

iPS-MSCs (5 weeks of differentiation = 4 passages) and MSCs (P4) were seeded at a density of 2500 cells/cm^2 in 12-well plates. After 6 days, cell numbers were counted with a Neubauer chamber (Brand, Wertheim, Germany). Similar results were observed with Alamar Blue staining (data not shown). For analysis of cumulative population doublings (cPDs), cells were counted at each passage and reseeded at a density of 5,000 cells/cm^2. cPDs were calculated as described before (Cholewa et al., 2011).

Immunophenotypic analysis (Figure 1C and S1C)

Surface marker expression was analyzed on a FACS Canto II (BD, Franklin Lakes, NJ, USA). Antibodies used for immunophenotypic analysis: CD14-allophycocyanin (APC; clone M5E2; BD Biosciences), CD29-phycoerythrin (PE; clone MAR4; BD Biosciences), CD31-PE (clone WM59; BD Biosciences), CD34-APC (clone 581; BD Biosciences), CD45-APC (clone HI30; BD Biosciences), CD73-PE (clone AD2; BD Biosciences), CD90-APC (clone 5E10; BD Biosciences), CD105-fluorescein isothiocyanate (FITC; clone MEM-226; ImmunoTools, Friesoythe, Germany). Unstained cells served as autofluorescent control. For each sample, 10,000 events were measured with emission wavelengths of 519 nm (FITC), 578 nm (PE), and 660 nm (APC). Data was analyzed using WinMDI 2.9 software.

In vitro differentiation toward mesodermal lineages (Figure 1D and S1D)

Adipogenic, osteogenic, and chondrogenic differentiation of MSCs (passage 7) and iPS-MSCs (5 weeks of differentiation) was induced as described before (Koch et al., 2011; Wagner et al., 2008). Briefly, cells were cultivated in adipogenic, osteogenic, or chondrogenic differentiation medium, respectively. After 21 days fat droplet formation upon adipogenic differentiation was analyzed by staining with BODIPY (4,4-difluoro-1,2,5,7,8-pentamethyl-4-bora-3a,4a-diaza-s-indacene; Invitrogen, Carlsbad, CA, USA) and counter-stained with DAPI (4′,6-Diamidin-2-phenylindol; Molecular Probes, Carlsbad, CA, USA). Osteogenic differentiation was analyzed by Alizarin Red (Sigma-Aldrich) staining. Chondrogenic differentiation was assessed in fixed pellets with Alcian Blue staining in combination with Periodic acid-Schiff (PAS).

Quantitative Real-Time(RT)-PCR for mesodermal lineage marker genes (Figure 1E)

Total RNA of differentiated and control cells was isolated with the NucleoSpin® miRNA Isolation Kit and analyzed with the NanoDrop ND1000 spectrometer. RNA was then converted into cDNA with the High-Capacity cDNA Reverse Transcription Kit (Applied Biosystems, Foster City, California, USA). Quantitative RT-PCR was performed using the TaqMan Gene Expression Master Mix in the StepOnePlus™ Real-Time PCR System (Applied Biosystems) according to the manufacturer’s instructions. Expression levels were normalized to *GAPDH*. Relative mRNA expression levels were calculated in relation to the corresponding undifferentiated control and fold ratio was determined using the $2^{\Delta\Delta Ct}$ values. The following TaqMan probes were used for adipogenic marker genes: *ADIPOQ* (adiponectin; Hs00605917_m1), *FABP4* (fatty acid binding protein 4; Hs01086177_m1); osteogenic marker genes: *RUNX2* (runt-related transcription factor 2; Hs00231692_m1), *SP7* (osterix; Hs01866874_s1), *COL1A1* (collagen, type 1, alpha 1; Hs00164004_m1), *SPARC* (osteonectin; Hs00234160_m1); chondrogenic marker genes: *SOX9* (sex determining region Y box 9; Hs00165814_m1), *ACAN* (aggrecan; Hs00153936_m1), *COL2A1* (collagen, type 2, alpha 1; Hs00264051_m1); reference gene: *GAPDH* (glyceraldehyde-3-phosphate dehydrogenase; Hs02758991_g1) (all Applied Biosystems).

Immunofluorescent staining (Figure S2A)

For staining of pluripotency markers, cells were cultured on cover slips coated with Matrigel or 0.1% gelatin and subsequently stained for OCT3/4, TRA-1-60, and SSEA4 as described previously (Willmann et al., 2013). Briefly, cells were fixed with 4% paraformaldehyde, blocked with goat serum, and subsequently stained with a primary and a fluorophore-conjugated secondary antibody (either Alexa594 or FITC). Cells were always counterstained with DAPI.

T cell proliferation assay (Figure 2G and 2H)

For analysis of immunosuppressive properties of MSCs and iPS-MSCs, mononuclear cells were isolated from whole blood of healthy donors after informed consent by Biocoll density gradient centrifugation (1.077 g/cm^3, Biochrom KG, Berlin, Germany). CD4$^+$ T cells were enriched using the CD4 MicroBead Kit on a MiniMACS system according to the manufacturer’s instructions (Miltenyi Biotec GmbH, Bergisch Gladbach, Germany). CD4$^+$ T cells were subsequently labeled with carboxyfluorescein succinidyl ester (CFSE; Sigma-Aldrich) to monitor cell divisions as described before (Walenda et al., 2010). T cells were stimulated with 1.5% phytohemagglutinin (PHA, Gibco) and IL-2 (3 ng/ml; PeproTech, Hamburg, Germany) as described before (Najar et al., 2009). T cells were cultivated in hPL Medium without or with the addition of MSCs or iPS-MSCs in different ratios (T cell:MSC ratios: 1:1; 5:1; 10:1). In each experiment, different MSC and iPSC preparations were used in parallel. As negative control, unstimulated T cells were used. After 5 days, T cells were stained with propidium iodide (BD Biosciences) to gate out dead cells and proliferation of T cells was assessed by the reduction in CFSE signal using flow cytometry on a FACS Canto II.

Gene expression analysis in Supplemental Information (Figure 2A-F, S2B-C and S3B-C)

Total RNA was isolated with the NucleoSpin® miRNA Isolation Kit (Macherey-Nagel, Düren, Germany) and analyzed using the Agilent 2100 Bioanalyzer (Agilent Technologies, Santa Clara, CA, USA) and the NanoDrop ND-1000 spectrometer (Thermo Scientific, Waltham, MA, USA). After hybridization on GeneChip Human Gene 1.0 ST Arrays (Affymetrix) raw data were normalized by RMA using Affymetrix Power Tools. All further calculations were performed on log2 transformed data. Significant genes were selected using a cutoff of at least 2-fold differential expression and an adjusted p-value smaller than 0.01 as determined by the eBayes method of the limma package in R.

For further bioinformatic analysis, Affymetrix IDs were matched to gene names using the getBM method of the biomaRT package (version 2.8.1). PluriTest analysis was done as described before (Müller et al., 2011)

Hierarchical clustering and pairwise correlation analysis were performed based on a Pearson correlation. GO-enrichment analysis for biological processes was performed using the topGO package in R (version 2.4.0) using Fisher's exact test. PhysiSpace analysis has been performed as described before (Lenz et al., 2013).

DNAm analysis in Supplemental Information (Figure 3, 4A-C, 4E-F, S3 and S4A-C)

Genomic DNA was isolated with the QIAamp DNA Blood Midi Kit (Qiagen, Hilden, Germany). DNA quality was assessed with a NanoDrop ND-1000 spectrometer and by gel electrophoresis and it was subsequently bisulfite-converted using the EZ DNA Methylation™ Kit (Zymo, Irvine, CA, USA). DNA was then hybridized on Infinium HumanMethylation450 BeadChips (Illumina). Hybridization and initial analysis of β-values (ranging from 0 [non-methylated] to 1 [100% methylated]) was performed with the BeadStudio Methylation Module at the DKFZ Gene Core Faciltity in Heidelberg, Germany. For further analysis we have only considered CpG sites on autosomes. Differentially methylated CpGs were selected by at least 20% difference in mean DNAm and adjusted p-values < 0.01 (limma package in R).

Density plots of DNAm level and unsupervised hierarchical clustering according to Euclidian Distance (with Ward´s Minimum Variance Method) were calculated with R. Affiliation of CpG sites to specific gene regions or CpG islands was used as described before (Sandoval et al., 2011). Selection of donor-specific CpGs (1,091 CpGs with high variation of DNAm in primary MSCs) (Shao et al., 2013) and tissue-specific CpGs (at least 15% difference in DNAm in MSCs from AT and BM) (Schellenberg et al., 2011) have been described in detail before. To estimate the state of cellular senescence we used the previously published Epigenetic-Senescence-Signature which is based on DNAm changes at six specific CpG sites - associated with the genes *GRM7, CASR, PRAMEF2, SELP, CASP14*, and *KRTAP13-3* (Koch et al., 2012; Koch and Wagner, 2013). DNAm at these specific CpG sites was analyzed by pyrosequencing (Varionostic GmbH, Ulm, Germany), and subsequent estimation of passage numbers and cPDs was performed with the online calculator which is accessible under http://www.molcell.rwth-aachen.de/dms/. Senescence-associated DNAm changes were furthermore calculated by the eBayes method of the limma package in R (based on M-values) with adjusted p-values < 0.01 and more than 20% change in DNAm level. To predict the donor-age based on DNAm profiles we either used a multivariate model based on 99 CpGs with age-associated DNAm changes in blood (Weidner et al., 2014), or a recently published age-predictor which has been generated for a broader range of tissues (Horvath, 2013) (http://128.97.66.147/horvath/age/).

Fibroblastoid colony-forming unit (CFU-f) assay (Figure 4D)

CFU-f frequency in long-term culture was determined by limiting dilution assay as described previously (Schellenberg et al., 2013; Schellenberg et al., 2012). This method can be used as a surrogate test for replicative senescence. Briefly, MSCs were seeded on a gelatin-coated 96-well plate at a density of 1, 3, 10, and 30 cells/well. 24 replicas for each density value were seeded. After 2 weeks of culture, cells were stained with Crystal Violet (Sigma-Aldrich) and the percentage of confluency was determined. Results were analyzed using the L-calc software available at http://www.stemcell.com/en/Products/All-Products/LCalc-Software.aspx.

Supplemental References

Chen,Y.S., Pelekanos,R.A., Ellis,R.L., Horne,R., Wolvetang,E.J., and Fisk,N.M. (2012). Small molecule mesengenic induction of human induced pluripotent stem cells to generate mesenchymal stem/stromal cells. Stem Cells Transl. Med *1*, 83-95.

Cholewa,D., Stiehl,T., Schellenberg,A., Bokermann,G., Joussen S, Koch C, Walenda T, Pallua,N., Marciniak-Czochra,A., Suschek,C.V., and Wagner W (2011). Expansion of adipose mesenchymal stromal cells is affected by human platelet lysate and plating density. Cell Transplant. *20*, 1409-1922.

Giuliani,M., Oudrhiri,N., Noman,Z.M., Vernochet,A., Chouaib,S., Azzarone,B., Durrbach,A., and Bennaceur-Griscelli,A. (2011). Human mesenchymal stem cells derived from induced pluripotent stem cells down-regulate NK-cell cytolytic machinery. Blood *118*, 3254-3262.

Hemeda,H., Kalz,J., Walenda,G., Lohmann,M., and Wagner,W. (2013). Heparin concentration is critical for cell culture with human platelet lysate. Cytotherapy *15*, 1174-1181.

Horn,P., Bokermann,G., Cholewa,D., Bork,S., Walenda,T., Koch,C., Drescher,W., Hutschenreuther,G., Zenke,M., Ho,A., and Wagner W (2010). Comparison of Individual Platelet Lysates for Isolation of Human Mesenchymal Stromal Cells. Cytotherapy *12*, 888-898.

Horvath,S. (2013). DNA methylation age of human tissues and cell types. Genome Biol *14*, R115.

Hynes,K., Menicanin,D., Mrozik,K.M., Gronthos,S., and Bartold,P.M. (2013). Generation of functional mesenchymal stem cells from different induced pluripotent stem cell lines. Stem Cells Dev *23*, 1084-1096.

Koch,C., Suschek,C.V., Lin Q, Bork S, Goergens M, Joussen S, Pallua,N., Ho A.D., Zenke M, and Wagner W (2011). Specific Age-associated DNA Methylation Changes in Human Dermal Fibroblasts. PLoS ONE *6*, e16679.

Koch,C.M., Joussen,S., Schellenberg,A., Lin,Q., Zenke,M., and Wagner,W. (2012). Monitoring of Cellular Senescence by DNA-Methylation at Specific CpG sites. Aging Cell *11*, 366-369.

Koch,C.M. and Wagner,W. (2013). Epigenetic Biomarker to Determine Replicative Senescence of Cultured Cells. Methods in Molecular Biology *1048*, 309-21.

Lenz,M., Schuldt,B.M., Muller,F.J., and Schuppert,A. (2013). PhysioSpace: relating gene expression experiments from heterogeneous sources using shared physiological processes. PLoS ONE *8*, e77627.

Lian,Q., Zhang,Y., Zhang,J., Zhang,H.K., Wu,X., Zhang,Y., Lam,F.F., Kang,S., Xia,J.C., Lai,W.H., Au,K.W., Chow,Y.Y., Siu,C.W., Lee,C.N., and Tse,H.F. (2010). Functional mesenchymal stem cells derived from human induced pluripotent stem cells attenuate limb ischemia in mice. Circulation *121*, 1113-1123.

Liu,J., Chen,W., Zhao,Z., and Xu,H.H. (2013). Reprogramming of mesenchymal stem cells derived from iPSCs seeded on biofunctionalized calcium phosphate scaffold for bone engineering. Biomaterials *34*, 7862-7872.

Miao,Q., Shim,W., Tee,N., Lim,S.Y., Chung,Y.Y., Ja,K.P., Ooi,T.H., Tan,G., Kong,G., Wei,H., Lim,C.H., Sin,Y.K., and Wong,P. (2014). iPSC-derived human mesenchymal stem cells improve myocardial strain of infarcted myocardium. J Cell Mol Med [epub ahead of print; doi: 10.1111/jcmm.12351].

Moslem,M., Valojerdi,M.R., Pournasr,B., Muhammadnejad,A., and Baharvand,H. (2013). Therapeutic potential of human induced pluripotent stem cell-derived mesenchymal stem cells in mice with lethal fulminant hepatic failure. Cell Transplant *22*, 1785-1799.

Müller,F.J., Schuldt,B.M., Williams,R., Mason,D., Altun,G., Papapetrou,E.P., Danner,S., Goldmann,J.E., Herbst,A., Schmidt,N.O., Aldenhoff,J.B., Laurent,L.C., and Loring,J.F. (2011). A bioinformatic assay for pluripotency in human cells. Nat Methods *8*, 315-317.

Najar,M., Rouas,R., Raicevic,G., Boufker,H.I., Lewalle,P., Meuleman,N., Bron,D., Toungouz,M., Martiat,P., and Lagneaux,L. (2009). Mesenchymal stromal cells promote or suppress the proliferation of T lymphocytes from cord blood and peripheral blood: the importance of low cell ratio and role of interleukin-6. Cytotherapy *11*, 570-583.

Sandoval,J., Heyn,H.A., Moran,S., Serra-Musach,J., Pujana,M.A., Bibikova,M., and Esteller,M. (2011). Validation of a DNA methylation microarray for 450,000 CpG sites in the human genome. Epigenetics *6*, 692-702.

Schellenberg,A., Hemeda,H., and Wagner,W. (2013). Tracking of Replicative Senescence in Mesenchymal Stem Cells by Colony-Forming Unit Frequency. Methods in Molecular Biology *976*, 143-154.

Schellenberg,A., Lin,Q., Schueler,H., Koch,C., Joussen,S., Denecke,B., Walenda,G., Pallua,N., Suschek,C., Zenke,M., and Wagner,W. (2011). Replicative senescence of mesenchymal stem cells causes DNA-methylation changes which correlate with repressive histone marks. Aging (Albany NY) *3*, 873-888.

Schellenberg,A., Stiehl,T., Horn,P., Joussen,S., Pallua,N., Ho,A., and Wagner W (2012). Population Dynamics of Mesenchymal Stromal Cells during Culture Expansion. Cytotherapy *14*, 401-411.

Shao,K., Koch,C.M., Gupta,M.K., Lin,Q., Lenz,M., Laufs,S., Denecke,B., Schmidt,M., Linke,M., Hennies,H.C., Hscheler,J., Zenke,M., Zechner,U., Šarić,T., and Wagner,W. (2013). Induced Pluripotent Mesenchymal Stromal Cell Clones Retain Donor-Derived Differences in DNA Methylation Profiles. Molecular Therapy *21*, 240-250.

Sun,Y.Q., Deng,M.X., He,J., Zeng,Q.X., Wen,W., Wong,D.S., Tse,H.F., Xu,G., Lian,Q., Shi,J., and Fu,Q.L. (2012). Human pluripotent stem cell-derived mesenchymal stem cells prevent allergic airway inflammation in mice. Stem Cells *30*, 2692-2699.

Takahashi,K., Tanabe,K., Ohnuki,M., Narita,M., Ichisaka,T., Tomoda,K., and Yamanaka,S. (2007). Induction of pluripotent stem cells from adult human fibroblasts by defined factors. Cell *131*, 861-872.

Tang,M., Chen,W., Liu,J., Weir,M.D., Cheng,L., and Xu,H.H. (2014). Human induced pluripotent stem cell-derived mesenchymal stem cell seeding on calcium phosphate scaffold for bone regeneration. Tissue Eng Part A *20*, 1295-1305.

TheinHan,W., Liu,J., Tang,M., Chen,W., Cheng,L., and Xu,H.H. (2013). Induced pluripotent stem cell-derived mesenchymal stem cell seeding on biofunctionalized calcium phosphate cements. Bone Res *4*, 371-384.

Wagner,W., Horn,P., Castoldi,M., Diehlmann,A., Bork,S., Saffrich,R., Benes,V., Blake,J., Pfister,S., Eckstein,V., and Ho,A.D. (2008). Replicative Senescence of Mesenchymal Stem Cells - a Continuous and Organized Process. PLoS ONE *5*, e2213.

Walenda,T., Bork,S., Horn,P., Wein,F., Saffrich,R., Diehlmann,A., Eckstein,V., Ho,A.D., and Wagner,W. (2010). Co-Culture with Mesenchymal Stromal Cells Increases Proliferation and Maintenance of Hematopoietic Progenitor Cells. J Cell Mol Med *14*, 337-350.

Weidner,C.I., Lin,Q., Koch,C.M., Eisele,L., Beier,F., Ziegler,P., Bauerschlag,D.O., Jockel,K.H., Erbel,R., Muhleisen,T.W., Zenke,M., Brummendorf,T.H., and Wagner,W. (2014). Aging of blood can be tracked by DNA methylation changes at just three CpG sites. Genome Biol *15*, R24.

Willmann,C.A., Hemeda,H., Pieper,L.A., Lenz,M., Qin,J., Joussen,S., Sontag,S., Wanek,P., Denecke,B., Schuler,H.M., Zenke,M., and Wagner,W. (2013). To clone or not to clone? Induced pluripotent stem cells can be generated in bulk culture. PLoS ONE *8*, e65324.
