# Supplementary material for: Epigenetic Rejuvenation of Mesenchymal Stromal Cells Derived from Induced Pluripotent Stem Cells
Source: Stem Cell Reports. 2014 Aug 14;3(3):414–22. doi: 10.1016/j.stemcr.2014.07.003 (PMC4266008; doi:10.1016/j.stemcr.2014.07.003)
Supplement: Document S2. Article plus Supplemental Information [file mmc5.pdf]

Stem Cell Reports
Report
OPEN ACCESS

Epigenetic Rejuvenation of Mesenchymal Stromal Cells Derived from Induced Pluripotent Stem Cells

Joana Frobel,[1] Hatim Hemeda,[1] Michael Lenz,[2] Giulio Abagnale,[1] Sylvia Joussen,[1] Bernd Denecke,[3] Tomo Šarić,[4] Martin Zenke,[1,5] and Wolfgang Wagner[1,*]
[1]Helmholtz-Institute for Biomedical Engineering, RWTH Aachen University Medical School, 52074 Aachen, Germany
[2]Aachen Institute for Advanced Study in Computational Engineering Science (AICES), RWTH Aachen University, 52074 Aachen, Germany
[3]Interdisciplinary Center for Clinical Research (IZKF), RWTH Aachen University Medical School, 52074 Aachen, Germany
[4]Center for Physiology and Pathophysiology, Institute for Neurophysiology, University of Cologne, 50931 Cologne, Germany
[5]Institute for Biomedical Engineering, Cell Biology, RWTH Aachen University Medical School, 52074 Aachen, Germany
*Correspondence: wwagner@ukaachen.de
http://dx.doi.org/10.1016/j.stemcr.2014.07.003
This is an open access article under the CC BY license (http://creativecommons.org/licenses/by/3.0/).

SUMMARY

Standardization of mesenchymal stromal cells (MSCs) remains a major obstacle in regenerative medicine. Starting material and culture expansion affect cell preparations and render comparison between studies difficult. In contrast, induced pluripotent stem cells (iPSCs) assimilate toward a ground state and may therefore give rise to more standardized cell preparations. We reprogrammed MSCs into iPSCs, which were subsequently redifferentiated toward MSCs. These iPS-MSCs revealed similar morphology, immunophenotype, in vitro differentiation potential, and gene expression profiles as primary MSCs. However, iPS-MSCs were impaired in suppressing T cell proliferation. DNA methylation (DNAm) profiles of iPSCs maintained donor-specific characteristics, whereas tissue-specific, senescence-associated, and age-related DNAm patterns were erased during reprogramming. iPS-MSCs reacquired senescence-associated DNAm during culture expansion, but they remained rejuvenated with regard to age-related DNAm. Overall, iPS-MSCs are similar to MSCs, but they reveal incomplete reacquisition of immunomodulatory function and MSC-specific DNAm patterns—particularly of DNAm patterns associated with tissue type and aging.

INTRODUCTION

Mesenchymal stromal cells (MSCs) are heterogeneous cell preparations and only a small subpopulation often referred to as "mesenchymal stem cells" possesses multilineage differentiation potential (Dominici et al., 2006). MSC preparations are greatly affected by starting material, such as bone marrow (BM) or adipose tissue (AT), and cell-culture media. Furthermore, they acquire functional changes during culture expansion ending in replicative senescence (Wagner and Ho, 2007). So far, MSCs are scarcely defined by fibroblastoid plastic adherent growth, a panel of nonspecific surface markers, and their capacity to differentiate toward adipogenic, osteogenic, and chondrogenic lineages (Dominici et al., 2006).

In this regard, induced pluripotent stem cells (iPSCs) converge to a better-defined ground state of pluripotency (Hackett et al., 2013). They can be differentiated into all cell types of the organism and—while in pluripotent state—cultured virtually indefinitely without signs of replicative senescence. Epigenetic profiles, such as DNA methylation (DNAm) patterns, are reorganized during reprogramming of somatic cells into iPSCs and closely resemble those of embryonic stem cells (ESCs) (Huang et al., 2014). In particular, senescence-associated DNAm, which is acquired during in vitro expansion (Koch et al., 2013), and age-related DNAm, which accumulate during aging of the organism (Horvath, 2013), are reversed to ground state. In comparison to primary cells, iPSCs are therefore better defined and offer a good starting point for large-scale generation of standardized derivatives, such as iPSC-derived MSCs (iPS-MSCs).

Several groups described strategies to derive MSC-like cells from either ESCs (Barberi et al., 2005; Boyd et al., 2009) or iPSCs (Liu et al., 2012; Diederichs and Tuan, 2014; Zhang et al., 2011). These approaches were based on coculture with primary MSCs, growth factor combinations, or spontaneous differentiation in embryoid bodies (EBs). So far, it has not been analyzed whether DNAm patterns of iPS-MSCs resemble those of primary MSCs.

RESULTS

Redifferentiation of iPSCs toward iPS-MSCs

We have recently reprogrammed MSCs from human bone marrow into iPSCs (Shao et al., 2013). These iPSCs were now redifferentiated toward iPS-MSCs using two alternative protocols: (1) the culture medium was simply exchanged to initial MSC-culture medium that comprised 10% human platelet lysate (hPL) or (2) iPSCs were allowed initially to differentiate into EBs in ultralow attachment plates for 7 days in differentiation medium (Figure S1A available online). Thereafter, cells were cultured under

standard culture conditions for MSCs with 10% hPL. After 35 days (four passages), the cells revealed a typical fibroblastoid growth pattern; these cells are referred to as iPS-MSCs in this manuscript (Figure 1A). iPS-MSCs passaged on gelatin-coated (Figure 1B) or noncoated (Figure S1B) tissue culture plastic exhibited significantly higher proliferation rates than primary MSCs of the corresponding passage. The immunophenotype of iPS-MSCs was essentially identical to primary MSCs ($CD29^+$, $CD73^+$, $CD90^+$, $CD105^+$, $CD14^-$, $CD31^-$, $CD34^-$, and $CD45^-$), albeit CD105 was less expressed in iPS-MSCs (Figures 1C and S1C). Furthermore, differentiation of iPS-MSCs toward osteogenic and chondrogenic lineage was equivalent to MSCs. Adipogenic differentiation was also induced in iPS-MSCs, although accumulation of lipid droplets was less pronounced than in primary MSCs (Figures 1D and S1D). These results on in vitro differentiation potential were further validated by upregulation of lineage-specific marker genes (Figure 1E). Taken together, iPS-MSCs fulfilled the minimal criteria for definition of MSCs (Dominici et al., 2006) - even though less prone to adipogenic differentiation. Because both redifferentiation protocols (with or without EB formation) did not reveal significant differences, we used the one-step differentiation protocol without EB formation and with gelatin coating for subsequent experiments.

iPS-MSCs Reveal Similar Gene Expression as MSCs

Global gene expression was compared in MSCs, iPSCs, and iPS-MSCs. Hierarchical cluster analysis revealed close relationship between iPS-MSCs and MSCs (Figure 2A), which was confirmed by pairwise correlation coefficients (Figure 2B). Gradual changes in gene expression were already observed during the first week of differentiation (Table S1): MSC marker genes including ecto-5′-nucleotidase (*NT5E;* CD73), CD44 antigen (*CD44*), alanyl aminopeptidase (*ANPEP*; CD13), and neural cell adhesion molecule 1 (*NCAM1*; CD56) were already upregulated. On the other hand, pluripotency genes were rapidly downregulated upon differentiation toward iPS-MSCs (Figures 2C, S2A, and S2B). Mesodermal genes typically expressed in MSCs were expressed at a similar level in iPS-MSCs (Figure 2D). Pairwise comparison of gene expression in MSCs, iPSCs, and iPS-MSCs revealed relatively few significantly differentially expressed genes between MSCs and iPS-MSCs (2-fold differential expression and adjusted p value <0.01; Figure 2E; Table S2): 339 genes were higher expressed in iPS-MSCs, and these were particularly enriched in gene ontology (GO) categories of transcriptional regulation, cell adhesion, and development; 214 genes were higher expressed in MSCs that were particularly enriched in GO categories for T cell activation and immune response (Figure 2F). Therefore, we used a surrogate assay to determine suppression of T cell proliferation in coculture with iPS-MSCs or MSCs. Indeed, MSCs significantly suppressed T cell proliferation in a dose-dependent manner, whereas this was not observed in iPS-MSCs, indicating lower immunomodulatory function (Figure 2G). To further classify gene expression profiles of iPS-MSCs, we used PhysioSpace analysis, a bioinformatics tool to interpret gene expression differences between two distinct cell types in terms of physiologically relevant expression patterns (Lenz et al., 2013) that provided further evidence that iPS-MSCs converged toward MSCs (Figure S2C). Overall, gene expression profiles supported the notion that iPS-MSCs closely resemble MSCs, even though there are differences in their immune function.

DNA Methylation Profiles of iPS-MSCs

Subsequently, we have analyzed DNAm profiles of MSCs, iPSCs, and iPS-MSCs (each of corresponding donors). Hierarchical clustering demonstrated that iPS-MSCs and MSCs cluster together (Figure 3A). At day 7 of differentiation toward iPS-MSCs the methylome was between pluripotent and nonpluripotent cells (Table S1). However, even after 5 weeks of differentiation 39,753 CpGs remained significantly differentially methylated between iPS-MSCs and MSCs (>20% differential DNAm; adjusted p value <0.01; Table S2), whereas only 13,896 CpGs reached this level of significance in iPS-MSCs versus iPSCs (Figure 3B). Overall, DNAm levels were higher in iPSCs and iPS-MSCs as compared to primary MSCs. Nevertheless, redifferentiation was associated with gradual loss of highly methylated and gain of unmethylated CpG sites (Figure S3A). DNAm was further analyzed in relevant genes – for example hypermethylation of POU class 5 homeobox 1 (*POU5F1*; OCT3/4) and Nanog homeobox gene (*NANOG*), and hypomethylation of surface marker genes *NT5E* (CD73*)* and endoglin (*ENG*; CD105) (Figure 3C). Notably, these DNAm patterns revealed high similarity between primary and redifferentiated MSCs in many genes, particularly in *NT5E*. Comparison of DNAm changes with expression changes of corresponding genes revealed some association, but there was no universal linear correlation (Figures S3B and S3C). Furthermore, DNAm differences of iPS-MSCs and MSCs were enriched in intergenic regions and shore regions of CpG islands (Figures 3D and S3D).

Comprehensive Analysis of DNAm Changes in iPS-MSCs

We have recently demonstrated that iPSCs maintain donor-specific characteristics in their DNAm pattern: 1,091 CpGs with the highest variation in different MSC preparations remained methylated at similar level in corresponding iPSCs (Shao et al., 2013). Here, we demonstrate that this donor-specific pattern was also maintained upon redifferentiation into iPS-MSCs (Figures 4A and S4A).

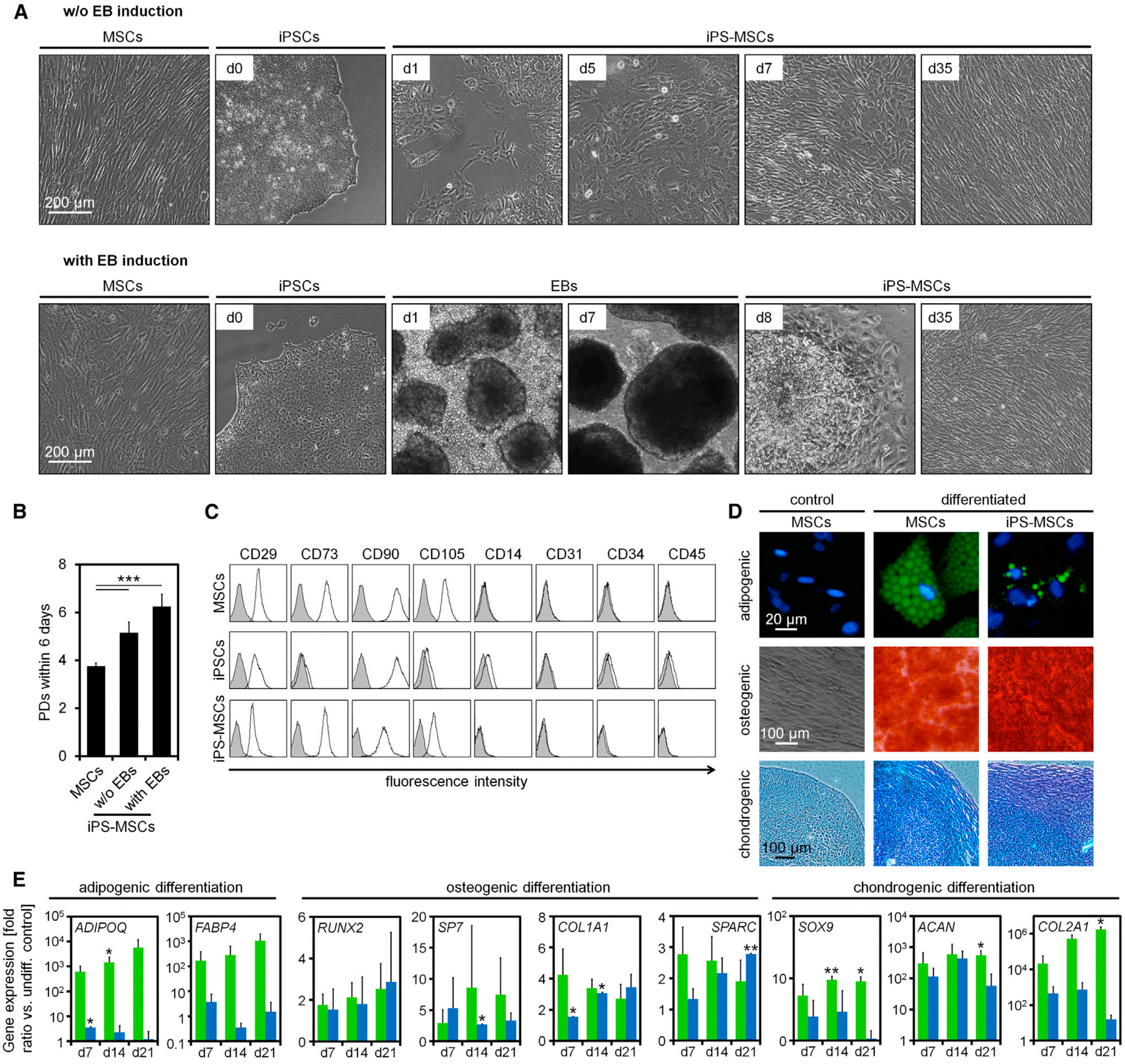

Figure 1. Generation of iPS-MSCs

(A) Phase contrast images of MSCs, iPSCs, and iPS-MSCs in the course of differentiation either with or without EB formation. Thirty-five days after induction of differentiation, iPS-MSCs revealed similar fibroblastoid morphology as MSCs.

(B) Population doublings (PDs) of MSCs and iPS-MSCs within 6 days of culture on gelatin-coated plates (N = 3; n = 3; mean ± SD; ***$p < 0.001$).

(C) iPS-MSCs displayed similar immunophenotypic characteristics as primary MSCs (autofluorescence is indicated in gray).

(D) MSCs and iPS-MSCs were differentiated toward adipogenic, osteogenic, or chondrogenic lineages for three weeks and subsequently stained with BODIPY/DAPI, alizarin red, or Alcian blue/PAS, respectively. Controls were simultaneously cultured in normal growth medium, and representative images are presented.

(E) In vitro differentiation potential was further assessed by quantitative real-time PCR of adipogenic (*ADIPOQ*, *FABP4*), osteogenic (*RUNX2*, *SP7*, *COL1A1*, *SPARC*), and chondrogenic (*SOX9*, *ACAN*, *COL2A1*) marker genes in MSCs (green) and iPS-MSCs (blue; N = 3; n = 2; mean ± SD; *$p < 0.05$; **$p < 0.01$ versus nondifferentiated control).

See also Figure S1.

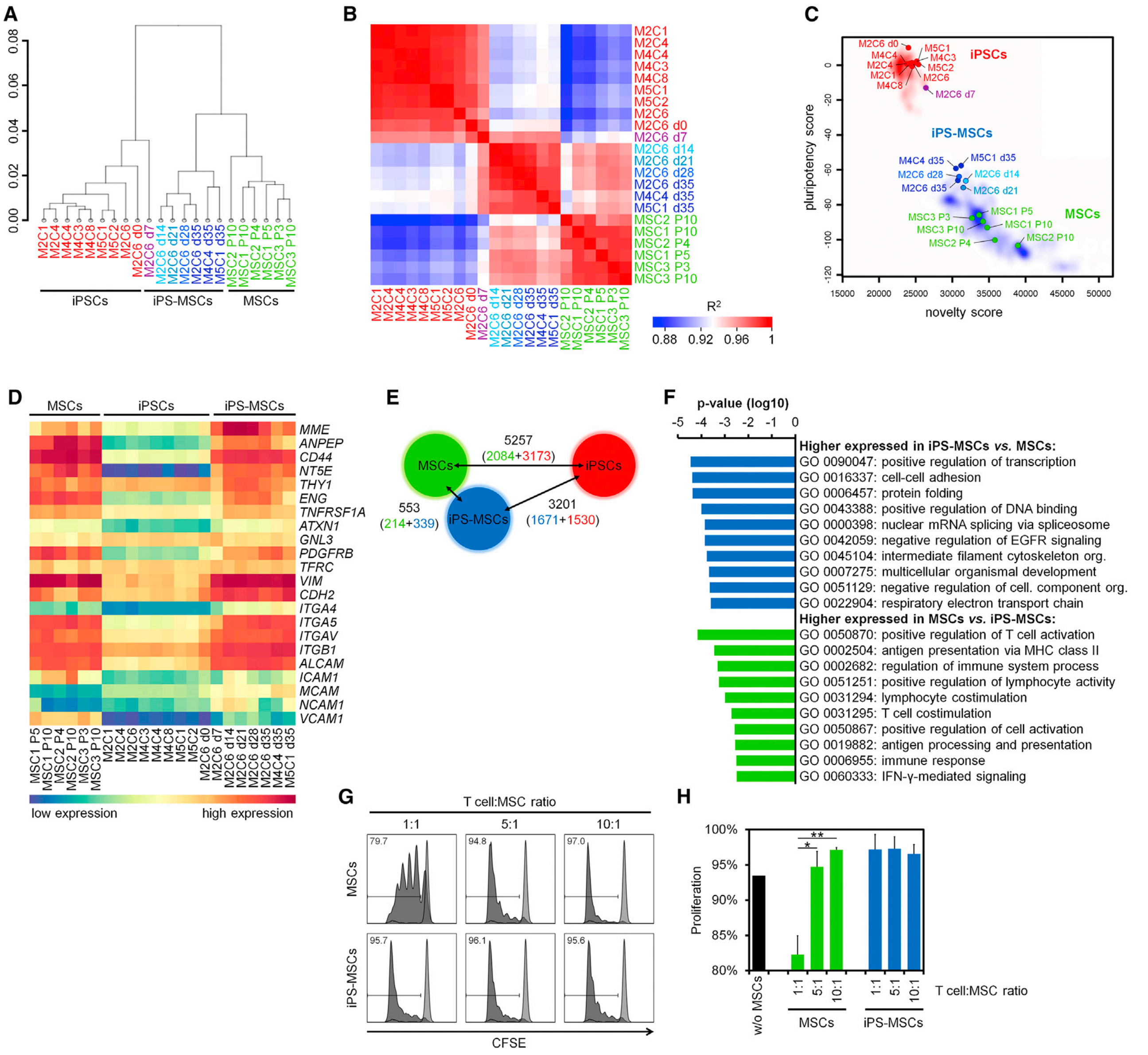

Figure 2. Gene Expression Profiles of iPS-MSCs Are Similar to Primary MSCs

(A) Hierarchical clustering revealed close relationship of iPS-MSCs and primary MSCs. MSC donor number ("M") and clone number ("C") are indicated for iPSCs and iPS-MSCs. Furthermore, passage numbers ("P") are provided for MSCs and time of redifferentiation ("d") for iPS-MSCs.
(B) Heatmap of pairwise correlation coefficients (R^2) demonstrates relationship of iPS-MSCs and MSCs.
(C) Pluripotency was assessed by PluriTest analysis (Müller et al., 2011). After differentiation for more than 7 days toward iPS-MSCs, cells were clearly associated with nonpluripotent samples (blue area) and not with pluripotent samples (red area; labeling of samples as in A).
(D) MSC marker genes were expressed at similar level in primary MSCs and iPS-MSCs.
(E) Number of differentially expressed genes between MSCs, iPSCs, and iPS-MSCs (>2-fold regulation; adjusted p value <0.01; for each cell type, the number of upregulated genes is indicated by color code).
(F) Gene ontology analysis of genes that are differentially expressed between MSCs and iPS-MSCs. The most significant categories are depicted.
(G) Activity of iPS-MSCs and MSCs on proliferation of stimulated $CD4^+$ T cells was assessed by flow cytometry and carboxyfluorescein succinimidyl ester (CFSE) staining. Different T cell:MSC ratios were used and representative histograms are depicted (unstimulated control is indicated in light gray). The percentage of proliferating cells is indicated in each histogram.
(H) Quantitative analysis of T cell proliferation assay was performed with percentage of proliferated cells as shown in (G) (MSCs: N = 3; iPS-MSCs: N = 2; mean ± SD; *$p < 0.05$; **$p < 0.01$).
See also Figure S2.

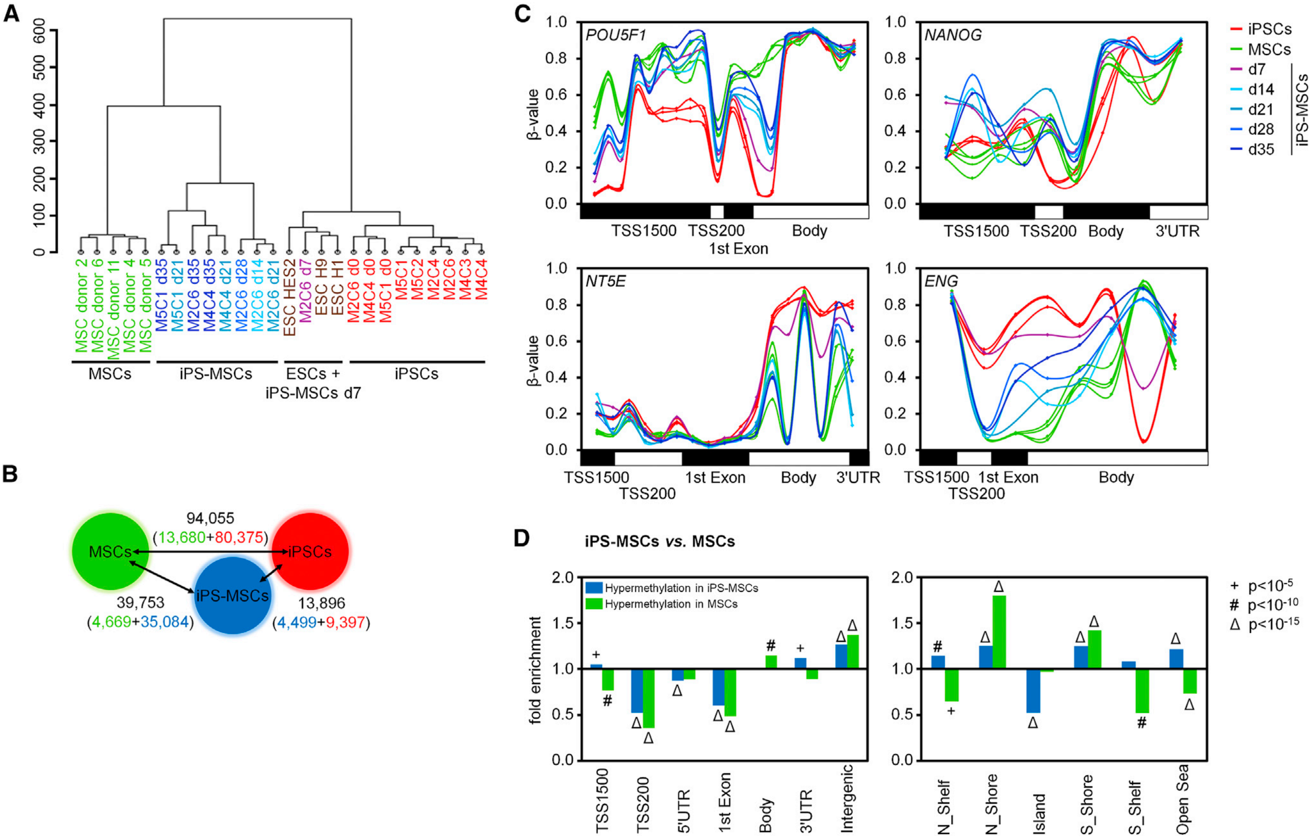

Figure 3. DNAm Profiles of iPS-MSCs

(A) Hierarchical clustering of global DNAm profiles.

(B) Number of CpGs with differential DNAm between MSCs, iPSCs, and iPS-MSCs (>20% change in DNAm level; adjusted p value <0.01; for each cell type hypermethylated CpGs are indicated by color code).

(C) DNAm levels (β values) of CpGs represented in the genes *POU5F1* (OCT3/4), *NANOG*, *NT5E* (CD73), and *ENG* (CD105) (TSS1500: 1,500 bp upstream of transcription start site; TSS200: 200 bp upstream of TSS; UTR).

(D) Enrichment of differential DNAm of MSCs versus iPS-MSCs in gene regions or in relation to CpG islands (p values were estimated by hypergeometric distribution).

See also Figure S3.

Subsequently, we analyzed if tissue-specific DNAm patterns are reestablished in iPS-MSCs. We have previously isolated MSCs from adipose tissue (AT) and bone marrow (BM); the latter were either derived from iliac crest (iliac) or caput femoris (hip): 1,711 CpGs revealed at least 15% differential DNAm in AT-MSCs versus BM-MSCs (Schellenberg et al., 2011). These DNAm changes were most significantly enriched in GO categories for nutrient level, lipid modification, and glucose metabolism reflecting functional differences of the originating tissues. Our MSCs from tibia plateau (knee) clustered with the other BM-MSCs. However, this tissue-specific DNAm pattern was erased by reprogramming and not reestablished upon differentiation toward iPS-MSCs (Figure 4B).

Long-term culture of MSCs is associated with highly reproducible DNAm changes—enriched in the homeobox gene cluster (*HOXB*) and the keratin associated protein (KRTAP) locus—which are almost entirely reversed in iPSCs (Koch et al., 2013). Here, we demonstrate that these senescence-associated DNAm changes are regained during culture expansion of iPS-MSCs. In particular CpGs, which are hypomethylated during expansion of MSCs, were reversed to high DNAm levels in iPSCs and subsequently again hypomethylated during expansion of iPS-MSCs (Figure S4B). Alternatively, we estimated cellular senescence by pyrosequencing of six senescence-associated CpGs (Koch et al., 2012). This Epigenetic-Senescence-Signature provides a biomarker that facilitates robust predictions for passage numbers (Figure S4C) and cumulative population doublings (Figures 4C and S4D): senescence predictions increased continuously during differentiation toward iPS-MSCs and after 35 days iPS-MSCs resembled MSCs of early passage in

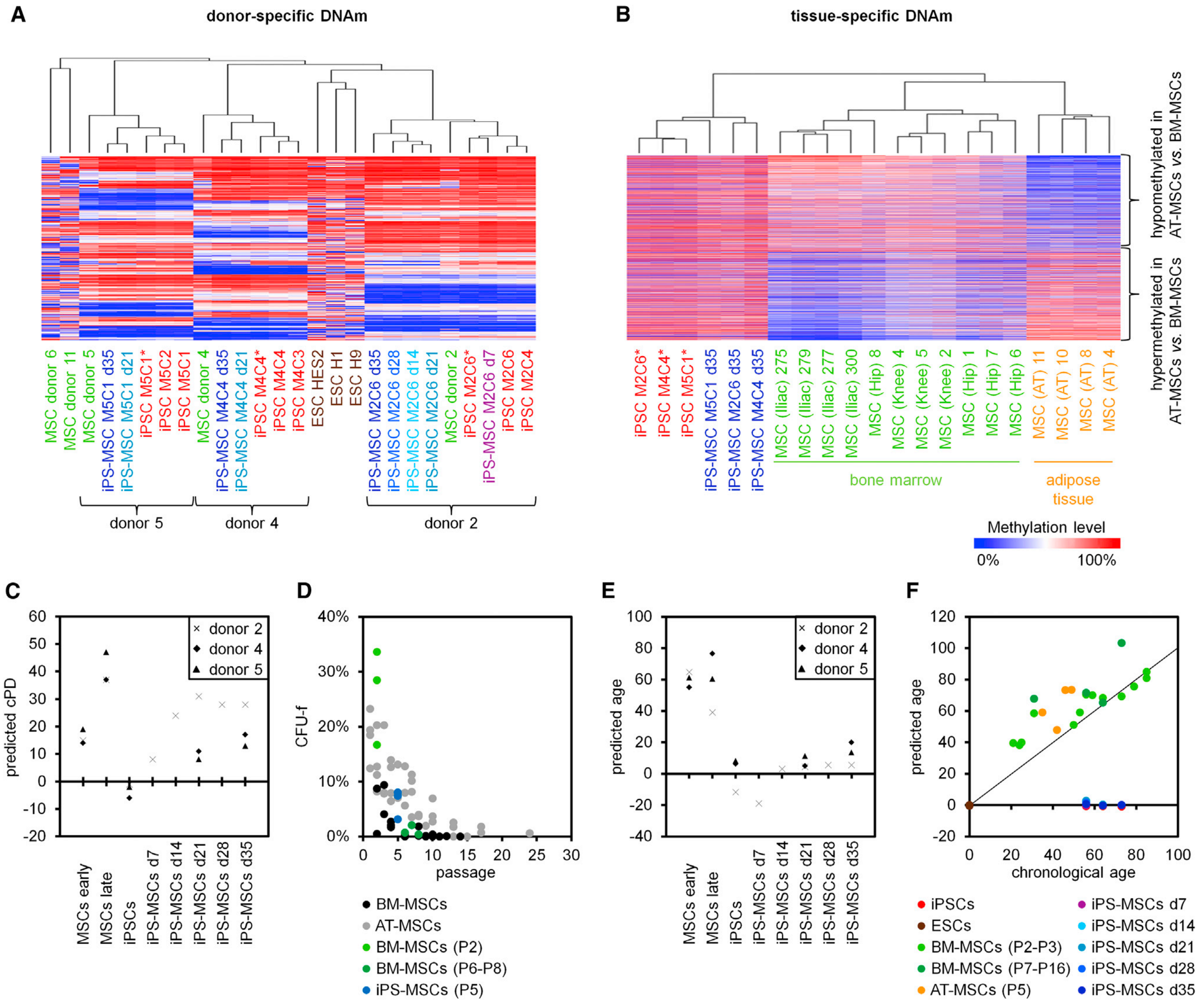

Figure 4. Donor-, Tissue-, and Age-Specific DNAm Changes

(A) Hierarchical cluster analysis of 1,091 CpGs with highest donor-specific variation in primary MSC preparations (SD > 0.2) (Shao et al., 2013) revealed that iPSCs and iPS-MSCs clustered with their parental cell preparations. This indicates that interindividual DNAm patterns are maintained in iPS-MSCs (*cultivated in mTeSR1).

(B) Hierarchical cluster analysis of 1,711 CpGs with differential DNAm in MSCs from adipose tissue (AT) and bone marrow (BM; >15% difference in mean methylation level) (Schellenberg et al., 2011) demonstrated that the BM-associated DNAm pattern is erased in iPSCs and not reestablished in iPS-MSCs.

(C) The state of cellular senescence was estimated by pyrosequencing analysis of six senescence-associated CpGs (Koch et al., 2012). Predictions of this Epigenetic-Senescence-Signature for cumulative population doublings (cPD) were reversed upon reprogramming into iPSCs and increased again during differentiation toward iPS-MSCs.

(D) To estimate the state of cellular senescence in iPS-MSCs we analyzed the frequency of fibroblastoid colony forming units (CFU-f). CFU-f frequency declines continuously in primary BM-MSCs and AT-MSCs (Schellenberg et al., 2012) and the number of CFU-f in iPS-MSCs after 35 days is in line with culture expansion for five passages.

(E) Donor age of cell preparations was estimated using a multivariate model based on DNAm of 99 age-related CpGs of blood (Weidner et al., 2014).

(F) Alternatively, donor age was predicted using a recently published predictor applicable for different tissues (Horvath, 2013). Overall, epigenetic rejuvenation upon reprogramming into iPSCs is also maintained in iPS-MSCs.

See also Figure S4.

their epigenetic makeup. To gain further insight into the state of cellular senescence of iPS-MSCs, we quantified the frequency of fibroblastoid colony-forming units (CFU-f), which rapidly declines during culture expansion of primary MSCs (Schellenberg et al., 2012). In iPS-MSCs at day 35 (passage 5), about 5% of the cells were capable to form colonies, which correspond to primary MSCs at passage 5 (Figure 4D).

Aging of the organism is also associated with specific DNAm changes, which are reversed upon reprogramming into iPSCs (Horvath, 2013; Weidner et al., 2014). Here, we analyzed whether these age-related DNAm changes are restored in iPS-MSCs. To this end, we have used a set of 99 CpGs, which reveal age-associated DNAm changes in blood. A multivariate model based on these CpGs has been used to estimate donor age (Weidner et al., 2014). In fact, iPS-MSCs reflect moderate accumulation of age-related DNAm but the samples were estimated much younger than the MSC donors (Figure 4E). Similar results were observed using the epigenetic predictor described by Horvath, which is based on more CpGs and which is applicable to different tissues and cell types (Horvath, 2013) (Figure 4F; correlation between the two predictors: R = 0.80). Overall, iPS-MSCs remained rejuvenated with regard to their DNAm profiles.

DISCUSSION

Induced pluripotent stem cells are a very good basis for generation of standardized cell types. However, differentiation to specific cell types remains a major challenge. Here, we describe a simple protocol for differentiation of iPSCs toward iPS-MSCs, which may be assisted by the fact that our iPSCs were initially derived from MSCs. Furthermore, the medium used for iPS-MSC induction is the same as for initial culture isolation of MSCs. These culture conditions would be compatible with guidelines of good manufacturing practice (GMP) in cellular therapy. Our iPS-MSCs fulfilled the minimal criteria of MSCs (Dominici et al., 2006), but the propensity for adipogenic differentiation was markedly decreased as compared to primary MSCs. This has also been observed by several other groups that used different protocols for generation of iPS-MSCs (Boyd et al., 2009; Chen et al., 2012; Diederichs and Tuan, 2014). Furthermore, adipogenic differentiation is also quite heterogeneous within primary MSCs (Schellenberg et al., 2012). Hence, a more sophisticated molecular definition is required for MSCs and for iPS-MSCs.

MSCs and iPS-MSCs revealed close relationship in gene expression profiles. However, genes associated with T cell activation and immune response were higher expressed in MSCs. Consistent with these observations, iPS-MSCs were impaired in suppressing T cell proliferation, indicating that iPS-MSCs have lower immunomodulatory properties than primary MSCs. Other authors indicated that ESC- and iPSC-derived MSCs are somewhat immunoprivileged and might even have therapeutic efficacy in autoimmune disorder models (de Peppo et al., 2010; Fu et al., 2012; Kimbrel et al., 2014). Therefore, the immunomodulatory function of iPS-MSCs—which is critical for clinical application—deserves further analysis in future studies.

Gene expression changes are not necessarily reflected on DNAm level and vice versa. In fact, many recent studies demonstrated no general correlation of DNAm and gene expression (Wagner et al., 2014), despite the common perception of hypermethylation in promoter regions should entail downregulation of gene expression. Although our understanding of the functional relevance of specific DNAm changes is so far limited epigenetic profiles are very well suited to classify cell preparations: DNAm can be provided as absolute β values at single base resolution; it is relatively stable; less prone to growth conditions; and less influenced by subpopulations, which may highly overexpress subsets of genes. In this regard, the large number of differentially methylated CpGs indicates that iPS-MSCs are rather “MSC-like” than direct correlates of primary MSCs—this has to be taken into account, but it does not exclude that iPS-MSCs may be valuable tools for cellular therapy, too.

We have demonstrated that donor-specific DNAm patterns are maintained upon reprogramming into iPSCs (Shao et al., 2013), and these also remain upon redifferentiation into iPS-MSCs. Thus, there is some epigenetic memory after chromatin remodeling—whether these donor-specific DNAm patterns are functionally relevant remains to be elucidated. On the other hand, tissue-specific epigenetic differences were erased during reprogramming and not reestablished in iPS-MSCs. This may explain some of the epigenetic discrepancy of MSCs and iPS-MSCs.

DNAm changes that accumulate during in vitro culture of MSCs (Koch et al., 2013) are also induced at a similar level during culture expansion of iPS-MSCs—apparently starting with loss of the pluripotent state. In contrast, age-related DNAm, which accumulates during aging of the organism (Weidner et al., 2014; Horvath, 2013) remains overall reset in iPS-MSCs. Notably, epigenetic rejuvenation does not counteract mutations, which may accumulate during in vitro culture. So far, the functional relevance of age-related DNAm changes and the underlying mechanism are not known, but the finding that they remain reset in iPS-MSCs is interesting and encourages further comparison with MSCs from different aged donors in vitro and in vivo. If age-related modifications contribute to loss of regenerative potential this may suggest higher regenerative potential of iPS-MSCs, which may also be reflected by the higher proliferation rates of iPS-MSCs as compared to primary MSCs.

Taken together, we described a simple one-step protocol to redifferentiate MSC-derived iPSCs toward MSCs. These iPS-MSCs reveal similar morphology, immunophenotype, and in vitro differentiation potential as primary MSCs; yet, there are marked differences in DNAm profiles that can, at least partially, be attributed to persistent reset of tissue-specific and age-related DNAm changes. In this regard, iPS-MSCs seem to provide more standardized cell products than primary MSCs, but the therapeutic efficiency—particularly with regard to their immunomodulatory functions—needs to be critically assessed.

EXPERIMENTAL PROCEDURES

A detailed description of all materials and methods is presented in the Supplemental Experimental Procedures.

Generation of iPS-MSCs

MSCs were isolated from bone marrow and reprogrammed in to iPSCs as described previously (Shao et al., 2013) (Table S3). For redifferentiation of iPSCs toward iPS-MSCs, we used two alternative strategies: (1) medium was simply exchanged for MSC standard medium with 10% hPL for 7 days, and cells were then further passaged in culture wells with 0.1% gelatin (Sigma-Aldrich) or noncoated plates, or (2) EBs were generated for 7 days in ultralow attachment plates (Corning) and then cultured on either gelatin-coated or noncoated plates.

Gene Expression Analysis

Gene expression profiles were analyzed by GeneChip Human Gene 1.0 ST Array (Affymetrix).

DNA Methylation Analysis

DNAm profiles were analyzed using the Infinium HumanMethylation450 BeadChip (Illumina), which addresses 485,577 CpG dinucleotides at a single-nucleotide resolution.

Statistical Analysis

Results are provided as mean ± SD of at least three independent experiments if not otherwise stated, and Student's t test was adopted to estimate statistical significance. "N" indicates the number of independent experiments, whereas "n" provides the number of technical replicates within the same experiment.

ACCESSION NUMBERS

Gene expression and DNAm data have been deposited at NCBI's Gene Expression Omnibus (http://www.ncbi.nlm.nih.gov/geo/) under accession numbers GSE54766, GSE38806, GSE46019 (gene expression) and GSE54767, GSE34688, GSE26519, GSE17448 (DNAm).

SUPPLEMENTAL INFORMATION

Supplemental Information includes Supplemental Experimental Procedures, four figures, and three tables and can be found with this article online at http://dx.doi.org/10.1016/j.stemcr.2014.07.003.

ACKNOWLEDGMENTS

We are grateful to Matthias Schick (gene core facility at the DKFZ, Heidelberg, Germany) for array hybridization and initial data analysis and to Qiong Lin (Institute for Biomedical Engineering, Cell Biology, RWTH, Aachen University Medical School, Germany) for analysis of aging signatures. This work was supported by the StemCellFactory consortium, cofunded by the European Union (European Regional Development Fund – Investing in your future) and German Federal State of North Rhine-Westphalia (M.Z., W.W., M.L.) and by the Stem Cell Network NRW (T.Š., W.W.); by the German Research Foundation (W.W.: WA 1706/3-2 and WA 1706/2-1; M.Z.: ZE 432/5-2 and ZE 432/6-1), and by the Else-Kröner Fresenius Stiftung (T.Š., W.W.). W.W. is a founder of Cygenia GmbH and member of its scientific advisory board.

Received: February 21, 2014
Revised: July 7, 2014
Accepted: July 9, 2014
Published: August 14, 2014

REFERENCES

Barberi, T., Willis, L.M., Socci, N.D., and Studer, L. (2005). Derivation of multipotent mesenchymal precursors from human embryonic stem cells. PLoS Med. *2*, e161.

Boyd, N.L., Robbins, K.R., Dhara, S.K., West, F.D., and Stice, S.L. (2009). Human embryonic stem cell-derived mesoderm-like epithelium transitions to mesenchymal progenitor cells. Tissue Eng. Part A *15*, 1897–1907.

Chen, Y.S., Pelekanos, R.A., Ellis, R.L., Horne, R., Wolvetang, E.J., and Fisk, N.M. (2012). Small molecule mesengenic induction of human induced pluripotent stem cells to generate mesenchymal stem/stromal cells. Stem Cells Transl. Med. *1*, 83–95.

de Peppo, G.M., Svensson, S., Lennerås, M., Synnergren, J., Stenberg, J., Strehl, R., Hyllner, J., Thomsen, P., and Karlsson, C. (2010). Human embryonic mesodermal progenitors highly resemble human mesenchymal stem cells and display high potential for tissue engineering applications. Tissue Eng. Part A *16*, 2161–2182.

Diederichs, S., and Tuan, R.S. (2014). Functional Comparison of Human-Induced Pluripotent Stem Cell-Derived Mesenchymal Cells and Bone Marrow-Derived Mesenchymal Stromal Cells from the Same Donor. Stem Cells Dev. *23*, 1594–1610.

Dominici, M., Le Blanc, K., Mueller, I., Slaper-Cortenbach, I., Marini, F., Krause, D., Deans, R., Keating, A., Prockop, Dj., and Horwitz, E. (2006). Minimal criteria for defining multipotent mesenchymal stromal cells. The International Society for Cellular Therapy position statement. Cytotherapy *8*, 315–317.

Fu, Q.L., Chow, Y.Y., Sun, S.J., Zeng, Q.X., Li, H.B., Shi, J.B., Sun, Y.Q., Wen, W., Tse, H.F., Lian, Q., and Xu, G. (2012). Mesenchymal stem cells derived from human induced pluripotent stem cells modulate T-cell phenotypes in allergic rhinitis. Allergy *67*, 1215–1222.

Hackett, J.A., Dietmann, S., Murakami, K., Down, T.A., Leitch, H.G., and Surani, M.A. (2013). Synergistic Mechanisms of DNA Demethylation during Transition to Ground-State Pluripotency. Stem Cell Rep. *1*, 518–531.

Horvath, S. (2013). DNA methylation age of human tissues and cell types. Genome Biol. *14*, R115.

Huang, K., Shen, Y., Xue, Z., Bibikova, M., April, C., Liu, Z., Cheng, L., Nagy, A., Pellegrini, M., Fan, J.B., and Fan, G. (2014). A panel of CpG methylation sites distinguishes human embryonic stem cells and induced pluripotent stem cells. Stem Cell Rep. *2*, 36–43.

Kimbrel, E.A., Kouris, N.A., Yavanian, G.J., Chu, J., Qin, Y., Chan, A., Singh, R.P., McCurdy, D., Gordon, L., Levinson, R.D., and Lanza, R. (2014). Mesenchymal Stem Cell Population Derived from Human Pluripotent Stem Cells Displays Potent Immunomodulatory and Therapeutic Properties. Stem Cells Dev. *23*, 1611–1624.

Koch, C.M., Joussen, S., Schellenberg, A., Lin, Q., Zenke, M., and Wagner, W. (2012). Monitoring of cellular senescence by DNA-methylation at specific CpG sites. Aging Cell *11*, 366–369.

Koch, C.M., Reck, K., Shao, K., Lin, Q., Joussen, S., Ziegler, P., Walenda, G., Drescher, W., Opalka, B., May, T., et al. (2013). Pluripotent stem cells escape from senescence-associated DNA methylation changes. Genome Res. *23*, 248–259.

Lenz, M., Schuldt, B.M., Müller, F.J., and Schuppert, A. (2013). PhysioSpace: relating gene expression experiments from heterogeneous sources using shared physiological processes. PLoS ONE *8*, e77627.

Liu, Y., Goldberg, A.J., Dennis, J.E., Gronowicz, G.A., and Kuhn, L.T. (2012). One-step derivation of mesenchymal stem cell (MSC)-like cells from human pluripotent stem cells on a fibrillar collagen coating. PLoS ONE *7*, e33225.

Müller, F.J., Schuldt, B.M., Williams, R., Mason, D., Altun, G., Papapetrou, E.P., Danner, S., Goldmann, J.E., Herbst, A., Schmidt, N.O., et al. (2011). A bioinformatic assay for pluripotency in human cells. Nat. Methods *8*, 315–317.

Schellenberg, A., Lin, Q., Schüler, H., Koch, C.M., Joussen, S., Denecke, B., Walenda, G., Pallua, N., Suschek, C.V., Zenke, M., and Wagner, W. (2011). Replicative senescence of mesenchymal stem cells causes DNA-methylation changes which correlate with repressive histone marks. Aging (Albany, N.Y. Online) *3*, 873–888.

Schellenberg, A., Stiehl, T., Horn, P., Joussen, S., Pallua, N., Ho, A.D., and Wagner, W. (2012). Population dynamics of mesenchymal stromal cells during culture expansion. Cytotherapy *14*, 401–411.

Shao, K., Koch, C., Gupta, M.K., Lin, Q., Lenz, M., Laufs, S., Denecke, B., Schmidt, M., Linke, M., Hennies, H.C., et al. (2013). Induced pluripotent mesenchymal stromal cell clones retain donor-derived differences in DNA methylation profiles. Mol. Ther. *21*, 240–250.

Wagner, W., and Ho, A.D. (2007). Mesenchymal stem cell preparations—comparing apples and oranges. Stem Cell Rev. *3*, 239–248.

Wagner, J.R., Busche, S., Ge, B., Kwan, T., Pastinen, T., and Blanchette, M. (2014). The relationship between DNA methylation, genetic and expression inter-individual variation in untransformed human fibroblasts. Genome Biol. *15*, R37.

Weidner, C.I., Lin, Q., Koch, C.M., Eisele, L., Beier, F., Ziegler, P., Bauerschlag, D.O., Jöckel, K.H., Erbel, R., Mühleisen, T.W., et al. (2014). Aging of blood can be tracked by DNA methylation changes at just three CpG sites. Genome Biol. *15*, R24.

Zhang, J., Lian, Q., Zhu, G., Zhou, F., Sui, L., Tan, C., Mutalif, R.A., Navasankari, R., Zhang, Y., Tse, H.F., et al. (2011). A human iPSC model of Hutchinson Gilford Progeria reveals vascular smooth muscle and mesenchymal stem cell defects. Cell Stem Cell *8*, 31–45.

Stem Cell Reports, Volume *3*

Supplemental Information

Epigenetic Rejuvenation of Mesenchymal Stromal Cells Derived from Induced Pluripotent Stem Cells

Joana Frobel, Hatim Hemeda, Michael Lenz, Giulio Abagnale, Sylvia Joussen, Bernd Denecke, Tomo Šarić, Martin Zenke, and Wolfgang Wagner

Table of Content

Figure S1. Generation and characterization of iPS-MSCs, related to Figure 1. **2**
Figure S2. Down-regulation of pluripotency genes in iPS-MSCs, related to Figure 2. **3**
Figure S3. Global DNAm changes upon re-differentiation, related to Figure 3. **4**
Figure S4. Donor-specific and senescence-associated DNAm in iPS-MSCs, related to Figure 4. **5**
Supplemental Experimental Procedures **6**
Culture of mesenchymal stromal cells 6
MSC-derived induced pluripotent stem cells 6
Generation of iPS-MSCs in Supplemental Information 6
Proliferation analysis (Figure 1B, S1B and S4D) 6
Immunophenotypic analysis (Figure 1C and S1C) 6
In vitro differentiation toward mesodermal lineages (Figure 1D and S1D) 7
Quantitative RT-PCR for mesodermal lineage marker genes (Figure 1E) 7
Immunofluorescent staining (Figure S2A) 7
T cell proliferation assay (Figure 2G and 2H) 7
Gene expression analysis in Supplemental Information (Figure 2A-F, S2B-C and S3B-C) 7
DNAm analysis in Supplemental Information (Figure 3, 4A-C, 4E-F, S3 and S4A-C) 8
Fibroblastoid colony-forming unit (CFU-f) assay (Figure 4D) 8
Supplemental References **8**

Table S1. Differential gene expression and DNA methylation in iPSCs vs. iPS-MSCs day 7 (separate file)
Table S2. Differential gene expression and DNA methylation in iPS-MSCs vs. MSCs (separate file)
Table S3. Information on MSCs used in this study (separate file)

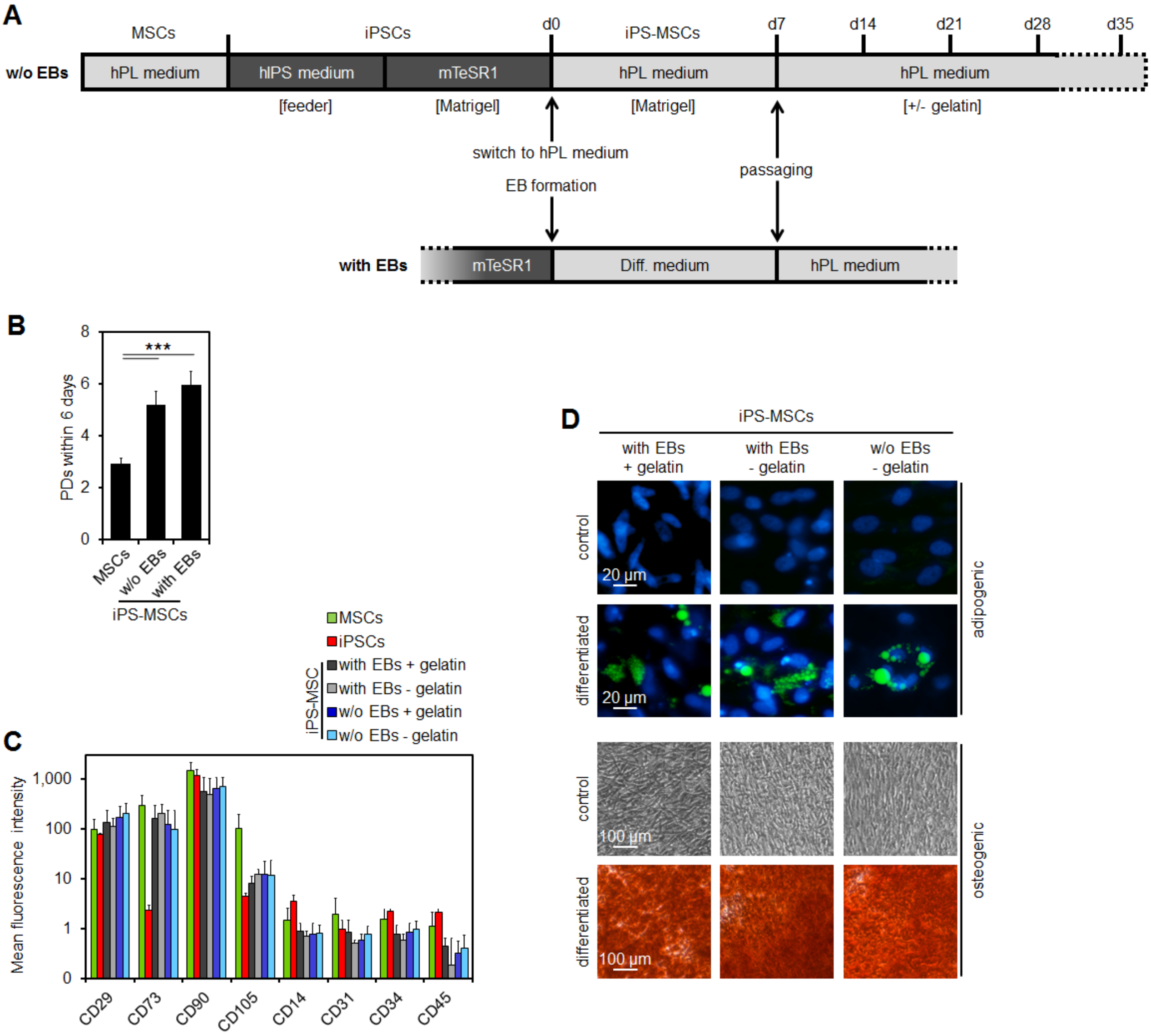

Figure S1. Generation and characterization of iPS-MSCs, related to Figure 1.

(A) Schematic representation of differentiation protocols towards iPS-MSCs. Human bone marrow-derived MSCs were reprogrammed into iPSCs, adopted to feeder-free growth conditions, and further differentiated towards iPS-MSCs using protocols either with or without embryoid body (EB) formation. Differentiation was induced with human platelet lysate (hPL) containing medium and iPS-MSCs were cultivated for five weeks with cell passages once per week.

(B) Population doublings (PDs) of iPS-MSCs and MSCs (both passage 4) on non-coated tissue culture plastic within 6 days (N = 3; n = 3; ± SD; *** $p < 0.001$).

(C) Flowcytometric comparison of MSCs, iPSCs, and iPS-MSCs. Quantitative analysis of data shown in Figure 1C is presented as mean fluorescence intensity normalized to autofluorescence (N ≥ 3; ± SD).

(D) Adipogenic and osteogenic differentiation of MSCs and iPS-MSCs was compared after three weeks of differentiation: osteogenic differentiation (lower panel) was very similar, whereas fat droplet formation in adipogenic differentiation of iPS-MSCs (upper panel) was less pronounced, irrespective of differentiation protocol.

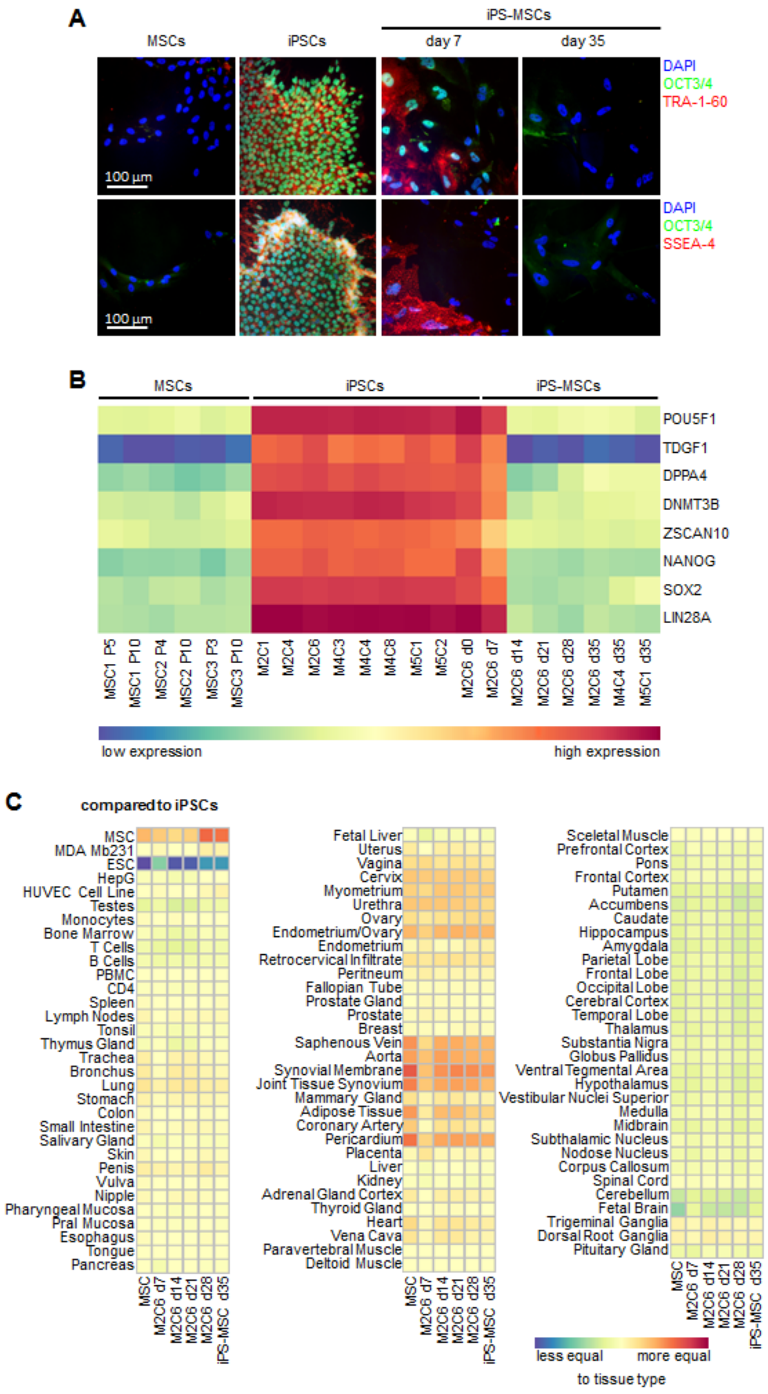

Figure S2. Down-regulation of pluripotency genes in iPS-MSCs, related to Figure 2.

(A) Pluripotency markers OCT3/4, TRA-1-60, and SSEA-4 were stained in MSCs, iPSCs, and iPS-MSCs. Seven days after differentiation towards iPS-MSCs there was still a mixed population of pluripotent and non-pluripotent cells. Size bars 100 µm.

(B) Heatmap of pluripotency gene expression (Affymetrix GeneChip analysis; M = MSC donor number; C = clone number; P = passage number; d = days of differentiation towards iPS-MSCs). Expression of pluripotency genes was down-regulated after 14 days of differentiation. *POU5F1* (OCT3/4); *TDGF1*: Teratocarcinoma-derived growth factor 1; *DPPA4*: Developmental pluripotency associated 4; *DNMT3B*: DNA (cytosine-5-) methyltransferase 3 beta; *ZSCAN10*: Zink finger protein 206; *NANOG*: Nanog; *SOX2*: Sex determining region Y box 2; *LIN28A*: Lin-28 homolog A.

(C) PhysioSpace analysis of MSCs and iPS-MSCs at different days of re-differentiation in comparison to iPSCs showed similarities (red) or differences (blue) to various tissue types. PhysioSpace is a bioinformatics method based on genome-wide tissue-specific gene expression patterns: differential gene expression of two distinct cell types is compared with tissue-specific expression patterns (Lenz et al., 2013). Notably, iPS-MSCs gradually became more MSC-like from day 7 to day 35 of re-differentiation, whereas similarities to ESC-patterns got lost. Furthermore, there was a prominent association of MSCs and iPS-MSCs to tissue-types containing high contents of connective-tissue, such as "Saphenous Vein", "Aorta", "Synovial Membrane", "Joint Tissue Synovium", or "Pericardium", as well as to "Adipose Tissue" which contains MSCs. Overall, MSCs and iPS-MSCs showed the same tissue-affiliations.

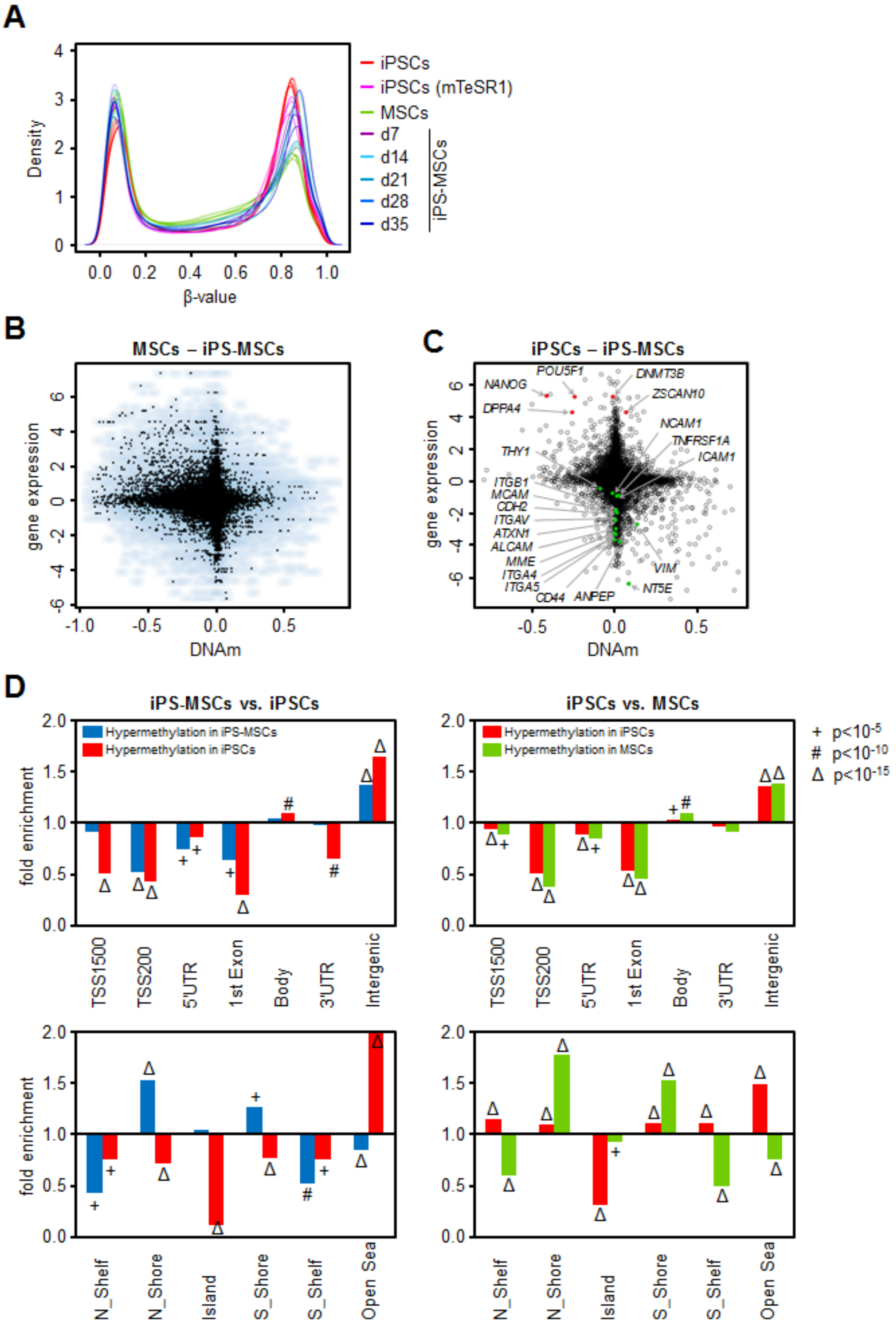

Figure S3. Global DNAm changes upon re-differentiation, related to Figure 3.

(A) Distribution of β-values over all CpG sites in MSCs, iPSCs, and iPS-MSCs. iPSCs were either cultivated on feeder cells (red) or in feeder-free conditions (mTeSR™1, pink).

(B) DNAm changes were analyzed in relation to gene expression changes of corresponding genes (MSCs *versus* iPS-MSCs). There was no clear negative correlation when considering all CpG sites (blue; Pearson correlation: -0.07) and only moderate association for CpG sites located within 200 base pairs up-stream of the transcription start site (TSS200; black dots; Pearson correlation: -0.16).

(C) Comparison of DNAm and gene expression of iPSCs *versus* iPS-MSCs demonstrated high negative correlation for several pluripotency associated genes (red: *NANOG*, *POU5F1*, *DPPA4*), whereas no negative correlation was observed for MSC marker genes (green). β-values of CpGs located within the TSS200 were averaged to be associated with each specific gene only once.

(D) Enrichment of DNAm changes in specific gene regions or in relation to CpG islands (either iPS-MSCs *versus* iPSCs, or iPSCs *versus* MSCs; p-values were estimated by hypergeometric distribution).

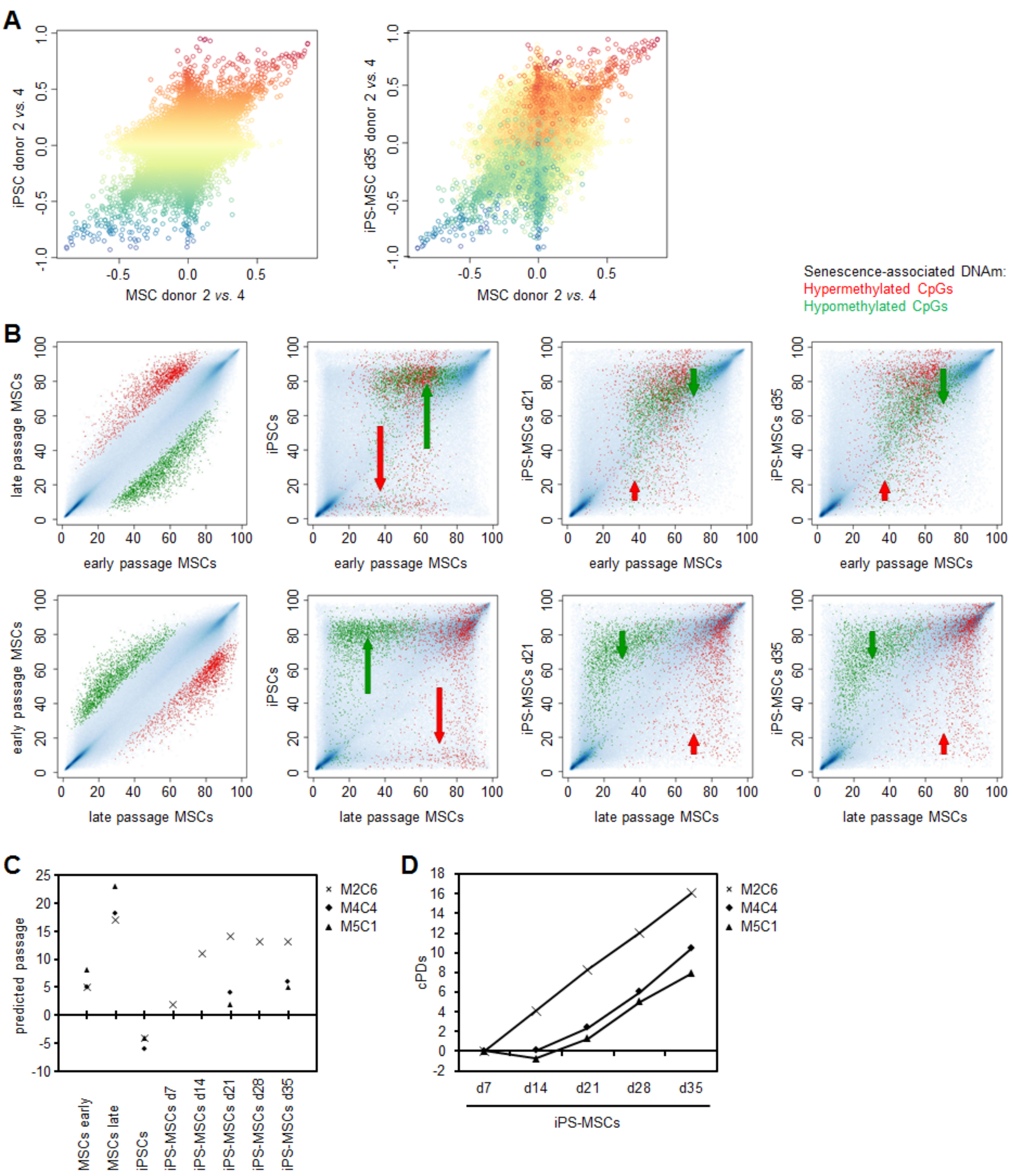

Figure S4. Donor-specific and senescence-associated DNAm in iPS-MSCs, related to Figure 4.

(A) Inter-individual comparison of DNAm profiles in MSCs *versus* iPSCs and MSCs *versus* iPS-MSCs (comparison of donors 2 and 4 is exemplarily depicted). Patient-specific DNAm patterns (left and right in the diagrams) were maintained upon reprogramming into iPSCs and also after re-differentiation towards iPS-MSCs. Furthermore, DNAm differences upon reprogramming (indicated by color code) were also maintained upon re-differentiation into iPS-MSCs.

(B) DNAm (numbers given in % methylation) of MSCs of early (P2-P5) *versus* late passage (P10) reveals CpGs which are hyper- or hypomethylated during culture expansion (depicted in red or green, respectively). DNAm at these senescence-associated CpGs is then presented in iPSCs or iPS-MSCs (either in comparison to MSCs of early passage (upper panel) or MSCs of late passage (lower panel)). Shifts in DNAm are indicated with red and green arrows, respectively.

(C) Cellular senescence was estimated by DNAm at six specific CpG sites using pyrosequencing (Koch et al., 2012). The results indicate that cellular senescence is reset in iPSCs and increased again during culture-expansion of iPS-MSCs.

(D) Cumulative population doublings (cPDs) of iPS-MSCs from three different donors (without EB-formation, with gelatin coating) were assessed upon induction of differentiation for 35 days. Overall, cPDs correlate with predictions for cPDs based on the Epigenetic-Senescence-Signature (Figure 4C).

Supplemental Experimental Procedures

Culture of mesenchymal stromal cells

MSCs were isolated from bone marrow (tibia plateau) of patients undergoing orthopedic surgery after written consent using guidelines approved by the Ethic Committee of the Use of Human Subjects at the University of Aachen (permit number: EK128/09). MSCs were culture-expanded in standard medium consisting of Dulbecco's Modified Eagle Medium (DMEM; 1 g/l glucose; PAA, Pasching, Austria), 1% L-glutamine (PAA), 1% penicillin/streptomycin (PAA), and 10% pooled human platelet lysate (hPL) as previously described (Horn et al., 2010). 0.1% heparin (5,000 IU/ml; Ratiopharm, Ulm, Germany) was added as anticoagulant (Hemeda et al., 2013). Upon 80% confluent growth, cells were reseeded at a density of 5,000 cells/cm^2. For comparison, we used data of adipose-tissue (AT) derived MSCs as described in detail before (Schellenberg et al., 2011; Schellenberg et al., 2012).

MSC-derived induced pluripotent stem cells

iPSCs were generated by infection of MSCs with retroviruses encoding the human transcription factors OCT3/4, SOX2, KLF4, and c-MYC as described previously (Takahashi et al., 2007). Briefly, MSCs were obtained from three different donors (2, 4, and 5) and infected at third passage with pMXs-based retroviruses (Addgene, Cambridge, MS, USA; (Shao et al., 2013). To exclude contamination of feeder cells, iPSCs were adjusted to a feeder-free system on matrigel (BD Biosciences, San Jose, CA, USA) in mTeSR™1 medium (Stemcell Technologies, Vancouver, BC, Canada) for at least three passages.

Generation of iPS-MSCs in Supplemental Information

Derivation of MSCs from iPSCs has been described by many groups using other procedures for differentiation (Chen et al., 2012; Giuliani et al., 2011; Hynes et al., 2013; Lian et al., 2010; Liu et al., 2013; Miao et al., 2014; Moslem et al., 2013; Sun et al., 2012; Tang et al., 2014; TheinHan et al., 2013). In this study, two differentiation strategies for re-differentiation of iPSCs towards iPS-MSCs were used:

Without EBs: Induction of iPS-MSCs was performed by a simple medium switch from mTeSR™1 medium to standard hPL medium. Cells were cultivated for 7 days on Matrigel, and then passaged into gelatin-coated or non-coated plates.

With EBs: For generation of EBs, iPSCs were detached with collagenase and transferred into ultra-low attachment plates. EBs were cultured for 7 days in differentiation medium consisting of Knockout-DMEM (Gibco, Carlsbad, CA, USA) supplemented with 20% FCS (Lonza, Basel, Switzerland), 1% L-glutamine, 1% penicillin/streptomycin, and 0.1 mM β-mercaptoethanol (all Gibco). Subsequently, EBs were seeded into culture plates either coated with 0.1% gelatin or without additional coating and cultured in standard hPL medium.

In the course of differentiation towards iPS-MSC the cells were passaged every 7 days with Trypsin-EDTA (Gibco), and reseeded in a density of 5,000 cells/cm^2.

Proliferation analysis (Figure 1B, S1B and S4D)

iPS-MSCs (5 weeks of differentiation = 4 passages) and MSCs (P4) were seeded at a density of 2500 cells/cm^2 in 12-well plates. After 6 days, cell numbers were counted with a Neubauer chamber (Brand, Wertheim, Germany). Similar results were observed with Alamar Blue staining (data not shown). For analysis of cumulative population doublings (cPDs), cells were counted at each passage and reseeded at a density of 5,000 cells/cm^2. cPDs were calculated as described before (Cholewa et al., 2011).

Immunophenotypic analysis (Figure 1C and S1C)

Surface marker expression was analyzed on a FACS Canto II (BD, Franklin Lakes, NJ, USA). Antibodies used for immunophenotypic analysis: CD14-allophycocyanin (APC; clone M5E2; BD Biosciences), CD29-phycoerythrin (PE; clone MAR4; BD Biosciences), CD31-PE (clone WM59; BD Biosciences), CD34-APC (clone 581; BD Biosciences), CD45-APC (clone HI30; BD Biosciences), CD73-PE (clone AD2; BD Biosciences), CD90-APC (clone 5E10; BD Biosciences), CD105-fluorescein isothiocyanate (FITC; clone MEM-226; ImmunoTools, Friesoythe, Germany). Unstained cells served as autofluorescent control. For each sample, 10,000 events were measured with emission wavelengths of 519 nm (FITC), 578 nm (PE), and 660 nm (APC). Data was analyzed using WinMDI 2.9 software.

In vitro differentiation toward mesodermal lineages (Figure 1D and S1D)

Adipogenic, osteogenic, and chondrogenic differentiation of MSCs (passage 7) and iPS-MSCs (5 weeks of differentiation) was induced as described before (Koch et al., 2011; Wagner et al., 2008). Briefly, cells were cultivated in adipogenic, osteogenic, or chondrogenic differentiation medium, respectively. After 21 days fat droplet formation upon adipogenic differentiation was analyzed by staining with BODIPY (4,4-difluoro-1,2,5,7,8-pentamethyl-4-bora-3a,4a-diaza-s-indacene; Invitrogen, Carlsbad, CA, USA) and counter-stained with DAPI (4′,6-Diamidin-2-phenylindol; Molecular Probes, Carlsbad, CA, USA). Osteogenic differentiation was analyzed by Alizarin Red (Sigma-Aldrich) staining. Chondrogenic differentiation was assessed in fixed pellets with Alcian Blue staining in combination with Periodic acid-Schiff (PAS).

Quantitative Real-Time(RT)-PCR for mesodermal lineage marker genes (Figure 1E)

Total RNA of differentiated and control cells was isolated with the NucleoSpin® miRNA Isolation Kit and analyzed with the NanoDrop ND1000 spectrometer. RNA was then converted into cDNA with the High-Capacity cDNA Reverse Transcription Kit (Applied Biosystems, Foster City, California, USA). Quantitative RT-PCR was performed using the TaqMan Gene Expression Master Mix in the StepOnePlus™ Real-Time PCR System (Applied Biosystems) according to the manufacturer's instructions. Expression levels were normalized to *GAPDH*. Relative mRNA expression levels were calculated in relation to the corresponding undifferentiated control and fold ratio was determined using the $2^{\Delta\Delta Ct}$ values. The following TaqMan probes were used for adipogenic marker genes: *ADIPOQ* (adiponectin; Hs00605917_m1), *FABP4* (fatty acid binding protein 4; Hs01086177_m1); osteogenic marker genes: *RUNX2* (runt-related transcription factor 2; Hs00231692_m1), *SP7* (osterix; Hs01866874_s1), *COL1A1* (collagen, type 1, alpha 1; Hs00164004_m1), *SPARC* (osteonectin; Hs00234160_m1); chondrogenic marker genes: *SOX9* (sex determining region Y box 9; Hs00165814_m1), *ACAN* (aggrecan; Hs00153936_m1), *COL2A1* (collagen, type 2, alpha 1; Hs00264051_m1); reference gene: *GAPDH* (glyceraldehyde-3-phosphate dehydrogenase; Hs02758991_g1) (all Applied Biosystems).

Immunofluorescent staining (Figure S2A)

For staining of pluripotency markers, cells were cultured on cover slips coated with Matrigel or 0.1% gelatin and subsequently stained for OCT3/4, TRA-1-60, and SSEA4 as described previously (Willmann et al., 2013). Briefly, cells were fixed with 4% paraformaldehyde, blocked with goat serum, and subsequently stained with a primary and a fluorophore-conjugated secondary antibody (either Alexa594 or FITC). Cells were always counterstained with DAPI.

T cell proliferation assay (Figure 2G and 2H)

For analysis of immunosuppressive properties of MSCs and iPS-MSCs, mononuclear cells were isolated from whole blood of healthy donors after informed consent by Biocoll density gradient centrifugation (1.077 g/cm^3, Biochrom KG, Berlin, Germany). $CD4^+$ T cells were enriched using the CD4 MicroBead Kit on a MiniMACS system according to the manufacturer's instructions (Miltenyi Biotec GmbH, Bergisch Gladbach, Germany). $CD4^+$ T cells were subsequently labeled with carboxyfluorescein succinidyl ester (CFSE; Sigma-Aldrich) to monitor cell divisions as described before (Walenda et al., 2010). T cells were stimulated with 1.5% phytohemagglutinin (PHA, Gibco) and IL-2 (3 ng/ml; PeproTech, Hamburg, Germany) as described before (Najar et al., 2009). T cells were cultivated in hPL Medium without or with the addition of MSCs or iPS-MSCs in different ratios (T cell:MSC ratios: 1:1; 5:1; 10:1). In each experiment, different MSC and iPSC preparations were used in parallel. As negative control, unstimulated T cells were used. After 5 days, T cells were stained with propidium iodide (BD Biosciences) to gate out dead cells and proliferation of T cells was assessed by the reduction in CFSE signal using flow cytometry on a FACS Canto II.

Gene expression analysis in Supplemental Information (Figure 2A-F, S2B-C and S3B-C)

Total RNA was isolated with the NucleoSpin® miRNA Isolation Kit (Macherey-Nagel, Düren, Germany) and analyzed using the Agilent 2100 Bioanalyzer (Agilent Technologies, Santa Clara, CA, USA) and the NanoDrop ND-1000 spectrometer (Thermo Scientific, Waltham, MA, USA). After hybridization on GeneChip Human Gene 1.0 ST Arrays (Affymetrix) raw data were normalized by RMA using Affymetrix Power Tools. All further calculations were performed on log2 transformed data. Significant genes were selected using a cutoff of at least 2-fold differential expression and an adjusted p-value smaller than 0.01 as determined by the eBayes method of the limma package in R.

For further bioinformatic analysis, Affymetrix IDs were matched to gene names using the getBM method of the biomaRT package (version 2.8.1). PluriTest analysis was done as described before (Müller et al., 2011)

Hierarchical clustering and pairwise correlation analysis were performed based on a Pearson correlation. GO-enrichment analysis for biological processes was performed using the topGO package in R (version 2.4.0) using Fisher's exact test. PhysiSpace analysis has been performed as described before (Lenz et al., 2013).

DNAm analysis in Supplemental Information (Figure 3, 4A-C, 4E-F, S3 and S4A-C)

Genomic DNA was isolated with the QIAamp DNA Blood Midi Kit (Qiagen, Hilden, Germany). DNA quality was assessed with a NanoDrop ND-1000 spectrometer and by gel electrophoresis and it was subsequently bisulfite-converted using the EZ DNA Methylation™ Kit (Zymo, Irvine, CA, USA). DNA was then hybridized on Infinium HumanMethylation450 BeadChips (Illumina). Hybridization and initial analysis of β-values (ranging from 0 [non-methylated] to 1 [100% methylated]) was performed with the BeadStudio Methylation Module at the DKFZ Gene Core Faciltity in Heidelberg, Germany. For further analysis we have only considered CpG sites on autosomes. Differentially methylated CpGs were selected by at least 20% difference in mean DNAm and adjusted p-values < 0.01 (limma package in R).

Density plots of DNAm level and unsupervised hierarchical clustering according to Euclidian Distance (with Ward´s Minimum Variance Method) were calculated with R. Affiliation of CpG sites to specific gene regions or CpG islands was used as described before (Sandoval et al., 2011). Selection of donor-specific CpGs (1,091 CpGs with high variation of DNAm in primary MSCs) (Shao et al., 2013) and tissue-specific CpGs (at least 15% difference in DNAm in MSCs from AT and BM) (Schellenberg et al., 2011) have been described in detail before. To estimate the state of cellular senescence we used the previously published Epigenetic-Senescence-Signature which is based on DNAm changes at six specific CpG sites - associated with the genes *GRM7, CASR, PRAMEF2, SELP, CASP14*, and *KRTAP13-3* (Koch et al., 2012; Koch and Wagner, 2013). DNAm at these specific CpG sites was analyzed by pyrosequencing (Varionostic GmbH, Ulm, Germany), and subsequent estimation of passage numbers and cPDs was performed with the online calculator which is accessible under http://www.molcell.rwth-aachen.de/dms/. Senescence-associated DNAm changes were furthermore calculated by the eBayes method of the limma package in R (based on M-values) with adjusted p-values < 0.01 and more than 20% change in DNAm level. To predict the donor-age based on DNAm profiles we either used a multivariate model based on 99 CpGs with age-associated DNAm changes in blood (Weidner et al., 2014), or a recently published age-predictor which has been generated for a broader range of tissues (Horvath, 2013) (http://128.97.66.147/horvath/age/).

Fibroblastoid colony-forming unit (CFU-f) assay (Figure 4D)

CFU-f frequency in long-term culture was determined by limiting dilution assay as described previously (Schellenberg et al., 2013; Schellenberg et al., 2012). This method can be used as a surrogate test for replicative senescence. Briefly, MSCs were seeded on a gelatin-coated 96-well plate at a density of 1, 3, 10, and 30 cells/well. 24 replicas for each density value were seeded. After 2 weeks of culture, cells were stained with Crystal Violet (Sigma-Aldrich) and the percentage of confluency was determined. Results were analyzed using the L-calc software available at http://www.stemcell.com/en/Products/All-Products/LCalc-Software.aspx.

Supplemental References

Chen,Y.S., Pelekanos,R.A., Ellis,R.L., Horne,R., Wolvetang,E.J., and Fisk,N.M. (2012). Small molecule mesengenic induction of human induced pluripotent stem cells to generate mesenchymal stem/stromal cells. Stem Cells Transl. Med *1*, 83-95.

Cholewa,D., Stiehl,T., Schellenberg,A., Bokermann,G., Joussen S, Koch C, Walenda T, Pallua,N., Marciniak-Czochra,A., Suschek,C.V., and Wagner W (2011). Expansion of adipose mesenchymal stromal cells is affected by human platelet lysate and plating density. Cell Transplant. *20*, 1409-1922.

Giuliani,M., Oudrhiri,N., Noman,Z.M., Vernochet,A., Chouaib,S., Azzarone,B., Durrbach,A., and Bennaceur-Griscelli,A. (2011). Human mesenchymal stem cells derived from induced pluripotent stem cells down-regulate NK-cell cytolytic machinery. Blood *118*, 3254-3262.

Hemeda,H., Kalz,J., Walenda,G., Lohmann,M., and Wagner,W. (2013). Heparin concentration is critical for cell culture with human platelet lysate. Cytotherapy *15*, 1174-1181.

Horn,P., Bokermann,G., Cholewa,D., Bork,S., Walenda,T., Koch,C., Drescher,W., Hutschenreuther,G., Zenke,M., Ho,A., and Wagner W (2010). Comparison of Individual Platelet Lysates for Isolation of Human Mesenchymal Stromal Cells. Cytotherapy *12*, 888-898.

Horvath,S. (2013). DNA methylation age of human tissues and cell types. Genome Biol *14*, R115.

Hynes,K., Menicanin,D., Mrozik,K.M., Gronthos,S., and Bartold,P.M. (2013). Generation of functional mesenchymal stem cells from different induced pluripotent stem cell lines. Stem Cells Dev *23*, 1084-1096.

Koch,C., Suschek,C.V., Lin Q, Bork S, Goergens M, Joussen S, Pallua,N., Ho A.D., Zenke M, and Wagner W (2011). Specific Age-associated DNA Methylation Changes in Human Dermal Fibroblasts. PLoS ONE *6*, e16679.

Koch,C.M., Joussen,S., Schellenberg,A., Lin,Q., Zenke,M., and Wagner,W. (2012). Monitoring of Cellular Senescence by DNA-Methylation at Specific CpG sites. Aging Cell *11*, 366-369.

Koch,C.M. and Wagner,W. (2013). Epigenetic Biomarker to Determine Replicative Senescence of Cultured Cells. Methods in Molecular Biology *1048*, 309-21.

Lenz,M., Schuldt,B.M., Muller,F.J., and Schuppert,A. (2013). PhysioSpace: relating gene expression experiments from heterogeneous sources using shared physiological processes. PLoS ONE *8*, e77627.

Lian,Q., Zhang,Y., Zhang,J., Zhang,H.K., Wu,X., Zhang,Y., Lam,F.F., Kang,S., Xia,J.C., Lai,W.H., Au,K.W., Chow,Y.Y., Siu,C.W., Lee,C.N., and Tse,H.F. (2010). Functional mesenchymal stem cells derived from human induced pluripotent stem cells attenuate limb ischemia in mice. Circulation *121*, 1113-1123.

Liu,J., Chen,W., Zhao,Z., and Xu,H.H. (2013). Reprogramming of mesenchymal stem cells derived from iPSCs seeded on biofunctionalized calcium phosphate scaffold for bone engineering. Biomaterials *34*, 7862-7872.

Miao,Q., Shim,W., Tee,N., Lim,S.Y., Chung,Y.Y., Ja,K.P., Ooi,T.H., Tan,G., Kong,G., Wei,H., Lim,C.H., Sin,Y.K., and Wong,P. (2014). iPSC-derived human mesenchymal stem cells improve myocardial strain of infarcted myocardium. J Cell Mol Med [epub ahead of print; doi: 10.1111/jcmm.12351].

Moslem,M., Valojerdi,M.R., Pournasr,B., Muhammadnejad,A., and Baharvand,H. (2013). Therapeutic potential of human induced pluripotent stem cell-derived mesenchymal stem cells in mice with lethal fulminant hepatic failure. Cell Transplant *22*, 1785-1799.

Müller,F.J., Schuldt,B.M., Williams,R., Mason,D., Altun,G., Papapetrou,E.P., Danner,S., Goldmann,J.E., Herbst,A., Schmidt,N.O., Aldenhoff,J.B., Laurent,L.C., and Loring,J.F. (2011). A bioinformatic assay for pluripotency in human cells. Nat Methods *8*, 315-317.

Najar,M., Rouas,R., Raicevic,G., Boufker,H.I., Lewalle,P., Meuleman,N., Bron,D., Toungouz,M., Martiat,P., and Lagneaux,L. (2009). Mesenchymal stromal cells promote or suppress the proliferation of T lymphocytes from cord blood and peripheral blood: the importance of low cell ratio and role of interleukin-6. Cytotherapy *11*, 570-583.

Sandoval,J., Heyn,H.A., Moran,S., Serra-Musach,J., Pujana,M.A., Bibikova,M., and Esteller,M. (2011). Validation of a DNA methylation microarray for 450,000 CpG sites in the human genome. Epigenetics *6*, 692-702.

Schellenberg,A., Hemeda,H., and Wagner,W. (2013). Tracking of Replicative Senescence in Mesenchymal Stem Cells by Colony-Forming Unit Frequency. Methods in Molecular Biology *976*, 143-154.

Schellenberg,A., Lin,Q., Schueler,H., Koch,C., Joussen,S., Denecke,B., Walenda,G., Pallua,N., Suschek,C., Zenke,M., and Wagner,W. (2011). Replicative senescence of mesenchymal stem cells causes DNA-methylation changes which correlate with repressive histone marks. Aging (Albany NY) *3*, 873-888.

Schellenberg,A., Stiehl,T., Horn,P., Joussen,S., Pallua,N., Ho,A., and Wagner W (2012). Population Dynamics of Mesenchymal Stromal Cells during Culture Expansion. Cytotherapy *14*, 401-411.

Shao,K., Koch,C.M., Gupta,M.K., Lin,Q., Lenz,M., Laufs,S., Denecke,B., Schmidt,M., Linke,M., Hennies,H.C., Hscheler,J., Zenke,M., Zechner,U., Šarić,T., and Wagner,W. (2013). Induced Pluripotent Mesenchymal Stromal Cell Clones Retain Donor-Derived Differences in DNA Methylation Profiles. Molecular Therapy *21*, 240-250.

Sun,Y.Q., Deng,M.X., He,J., Zeng,Q.X., Wen,W., Wong,D.S., Tse,H.F., Xu,G., Lian,Q., Shi,J., and Fu,Q.L. (2012). Human pluripotent stem cell-derived mesenchymal stem cells prevent allergic airway inflammation in mice. Stem Cells *30*, 2692-2699.

Takahashi,K., Tanabe,K., Ohnuki,M., Narita,M., Ichisaka,T., Tomoda,K., and Yamanaka,S. (2007). Induction of pluripotent stem cells from adult human fibroblasts by defined factors. Cell *131*, 861-872.

Tang,M., Chen,W., Liu,J., Weir,M.D., Cheng,L., and Xu,H.H. (2014). Human induced pluripotent stem cell-derived mesenchymal stem cell seeding on calcium phosphate scaffold for bone regeneration. Tissue Eng Part A *20*, 1295-1305.

TheinHan,W., Liu,J., Tang,M., Chen,W., Cheng,L., and Xu,H.H. (2013). Induced pluripotent stem cell-derived mesenchymal stem cell seeding on biofunctionalized calcium phosphate cements. Bone Res *4*, 371-384.

Wagner,W., Horn,P., Castoldi,M., Diehlmann,A., Bork,S., Saffrich,R., Benes,V., Blake,J., Pfister,S., Eckstein,V., and Ho,A.D. (2008). Replicative Senescence of Mesenchymal Stem Cells - a Continuous and Organized Process. PLoS ONE *5*, e2213.

Walenda,T., Bork,S., Horn,P., Wein,F., Saffrich,R., Diehlmann,A., Eckstein,V., Ho,A.D., and Wagner,W. (2010). Co-Culture with Mesenchymal Stromal Cells Increases Proliferation and Maintenance of Hematopoietic Progenitor Cells. J Cell Mol Med *14*, 337-350.

Weidner,C.I., Lin,Q., Koch,C.M., Eisele,L., Beier,F., Ziegler,P., Bauerschlag,D.O., Jockel,K.H., Erbel,R., Muhleisen,T.W., Zenke,M., Brummendorf,T.H., and Wagner,W. (2014). Aging of blood can be tracked by DNA methylation changes at just three CpG sites. Genome Biol *15*, R24.

Willmann,C.A., Hemeda,H., Pieper,L.A., Lenz,M., Qin,J., Joussen,S., Sontag,S., Wanek,P., Denecke,B., Schuler,H.M., Zenke,M., and Wagner,W. (2013). To clone or not to clone? Induced pluripotent stem cells can be generated in bulk culture. PLoS ONE *8*, e65324.
